# The Physiological Basis of Improved Heat Tolerance in Selected Emmer-Derived Hexaploid Wheat Genotypes

**DOI:** 10.3389/fpls.2021.739246

**Published:** 2021-10-07

**Authors:** Smi Ullah, Richard Trethowan, Helen Bramley

**Affiliations:** ^1^School of Life and Environmental Sciences, Plant Breeding Institute, Sydney Institute of Agriculture, The University of Sydney, Narrabri, NSW, Australia; ^2^School of Life and Environmental Sciences, Plant Breeding Institute, Sydney Institute of Agriculture, University of Sydney, Cobbitty, NSW, Australia

**Keywords:** emmer wheat, heat tolerance, physiological mechanism, heat chambers, genetic variation

## Abstract

Wheat is sensitive to high-temperature stress with crop development significantly impaired depending on the severity and timing of stress. Various physiological mechanisms have been identified as selection targets for heat tolerance; however, the complex nature of the trait and high genotype × temperature interaction limits the selection process. A three-tiered phenotyping strategy was used to overcome this limitation by using wheat genotypes developed from the ancient domesticated wheat, emmer (*Triticum dicoccon* Schrank), which was considered to have a wide variation for abiotic stress tolerance. A contrasting pair of emmer-based hexaploid lines (classified as tolerant; G1 and susceptible; G2) developed from a backcross to the same recurrent hexaploid parent was chosen based on heat stress responses in the field and was evaluated under controlled glasshouse conditions. The same pair of contrasting genotypes was also subsequently exposed to a short period of elevated temperature (4 days) at anthesis under field conditions using in-field temperature-controlled chambers. The glasshouse and field-based heat chambers produced comparable results. G1 was consistently better adapted to both extended and short periods of heat stress through slow leaf senescence under heat stress, which extended the grain filling period, increased photosynthetic capacity, increased grain filling rates, and resulted in greater kernel weight and higher yield. The use of a combination of phenotyping methods was effective in identifying heat tolerant materials and the mechanisms involved.

## Introduction

High temperature is a constraint to the sustainable production of wheat in major wheat growing areas of the world (Asseng et al., 2015). Heat stress can occur at any crop developmental stage depending on the growing region; however, the reproductive and grain filling stages are the most sensitive (Farooq et al., 2011; Barlow et al., 2015). Reproductive heat stress may cause pollen sterility, infertile ovules, decreased fertilization, and aborted florets (Prasad and Djanaguiraman, 2014), which ultimately decrease grain number and yield. Grain size is also reduced due to a shorter duration of the grain filling period and early senescence (Shirdelmoghanloo et al., 2016a), whereas grain quality decreases (Nuttall et al., 2017; Ullah et al., 2020a) and the percentage of shriveled/broken grain (also called screenings) increases (Farooq et al., 2011; Ferreira et al., 2012; Nuttall et al., 2017). Heat waves and higher average day/night temperatures are increasing with climate change; hence, mitigation strategies are needed to stabilize grain yield and quality (Prasad and Djanaguiraman, 2014; García et al., 2015).

Improving abiotic stress tolerance in wheat is constrained by limited genetic diversity due to traditional breeding processes and domestication (Nevo, 2014; Trethowan, 2014a). New sources of useful alleles must be found, and emmer wheat (*Triticum dicoccon* Schrank), an ancestral tetraploid wheat, can contribute to this diversity (Trethowan and Mujeeb-Kazi, 2008; Zaharieva et al., 2010; Ullah et al., 2018). Moreover, in our previous study (Ullah et al., 2020b), the most significant heat-tolerant trait contributed by emmer seemed to be stay-green. Stay-green refers to the retention of the green leaf area late in the season and delayed foliar senescence (Thomas and Ougham, 2014). The ability to “stay-green” has been related to increased rate of grain filling and duration, increased photosynthetic capacity, and higher yield in heat prone environments (Spano et al., 2003; Kumar et al., 2013; Pinto et al., 2016). Non-destructive methods, such as the measurement of canopy greenness using the normalized difference vegetation index (NDVI) and optically derived chlorophyll content, have been used to rapidly phenotype stay-green in the field (Lopes and Reynolds, 2012; Christopher et al., 2014; Talukder et al., 2014); however, stay-green has relatively low heritability, which has limited its adoption in breeding programs.

Accurate and relevant phenotyping methods are the keys to exploit genetic variation (Tardieu and Tuberosa, 2010; Chandrasekhar et al., 2017). Various phenotyping methods have been used for the evaluation of heat tolerance. However, a genotype-by-environment interaction can pose an obstacle to accurate phenotyping for heat tolerance although this can be reduced to some extent by managing the environmental conditions (Trethowan, 2014b). Nevertheless, a reverse strategy, which relies initially on field screening with later glasshouse confirmation, is more reliable and cost-effective (Telfer et al., 2018). A combination of screening methods may provide confirmation of the genotype responses and the mechanisms involved. In addition, although the effect of extended temperature stress (Lopes et al., 2012; Pinto et al., 2017) or heat shock (short periods of high temperature) have been investigated in wheat (Talukder et al., 2014; Shirdelmoghanloo et al., 2016a,b), hardly any studies have attempted to assess both aspects. The use of a combination of screening methods would enable the determination of superior heat-tolerant genotypes under both high temperature scenarios.

In our previous study, a large set of newly developed diverse emmer-based hexaploid wheat lines was sown under field conditions using a time-of-sowing (TOS) strategy to classify the material as heat susceptible or tolerant (Ullah et al., 2020b). A pair of lines, backcrossed once to the same recurrent bread wheat parent with equivalent high yield under optimum conditions but differing in yield under late sowing, was thus selected. To determine the probable mechanisms contributing to the yield differences under high temperature, these diverse but related emmer-derived hexaploid wheat genotypes were exposed to elevated temperature in a controlled glasshouse, which simulated the late sowing temperatures in the field from the heading stage or anthesis. We hypothesized that the favorable characteristics contributed by emmer, including stay-green, provided heat tolerance through improved photosynthetic capacity that supported better rates of grain filling. To test the effectiveness of the probable heat tolerance mechanism, we exposed the same pair of lines to short periods of high temperature at anthesis in the field using portable heat chambers. Using this strategy, we not only identified how emmer wheat contributed to heat tolerance in wheat but also confirmed that the trait improved yields under different high-temperature scenarios.

## Materials and Methods

### Plant Materials and Growth Conditions

#### Field Experiment

Plant material development is provided in detail in our previous paper (Ullah et al., 2018), and the field experimentation and environmental data are provided in detail by Ullah et al. (2020b). In summary, a set of 196 genotypes (11 parents and/or commercial check cultivars and 185 newly developed emmer-based hexaploid lines) was evaluated under field conditions as a part of our previous study (Ullah et al., 2020b). The list of the used plant materials is given in Supplementary Table 1. The field experiments were sown at the IA Watson Grains Research Centre, the University of Sydney, Narrabri, NSW, Australia during the cropping seasons of 2015 and 2016 in randomized complete block designs. Two adjacent experiments were sown each year at an optimal and a delayed sowing time and were referred to as TOS1 and TOS2, respectively. The TOS1 sowing date was mid-May, and TOS2 was sown 8 weeks later. Irrigation was used as required to limit drought stress.

#### Controlled Environment Experiment

Two contrasting emmer-derived wheat lines, G1 and G2, selected from the TOS experiments in the field were compared in a natural light glasshouse (Supplementary Table 1). The genotypes were selected for their similar phenology and yield in TOS1, but contrasting yield in TOS2.

The experiment was conducted in a glasshouse at the same research station as the field trial during 2016. Plants were grown in 5-L pots containing a commercial potting mixture (Premium potting mix, Searles, Kilcoy, South East Queensland, Australia) at day and night temperatures 20/14°C ± 2°C. Two seeds were sown per pot and then thinned to one plant at the two to three-leaf stage. Slow release fertilizer (N: P: K 19.4:1.6:5, Osmocote; Scotts Australia Pty. Ltd., Bella Vista, NSW, Australia) was applied at the rate of 1.3 g kg^−1^ potting mixture before planting. To provide consistent nutrition, a water-soluble fertilizer (N: P: K 23:4:18, Aquasol, Yates Australia, Padstow, NSW, Australia) was applied every 2 weeks up to anthesis at 2 g L^−1^ of water.

The controlled environment (CE) experiment consisted of three treatments:

CET1 = control = constant ambient conditions (20/14°C ± 2°C) until maturity,
CET2 = heat treatment (30/20°C ± 2°C) applied at heading (Z57) and maintained until maturity, and
CET3 = heat treatment (30/20°C ± 2°C) applied at anthesis (Z61) and maintained until maturity.

When the main stem of each plant reached the target growth stage, pots were moved into an adjacent room set at 30/20°C ± 2°C. These temperatures were chosen based on the maximum and minimum temperatures observed during heading, anthesis, and grain filling in the 2015 and 2016 field seasons (Supplementary Figure 1). All plants were irrigated two times daily to avoid the confounding effects of drought during heat treatment.

Each genotype × treatment (G × T) combination consisted of four replications for non-destructive measurements and four replications for destructive measurements at each time point. Pots were randomized within the glasshouse rooms and rotated frequently to avoid variation due to spatial arrangement.

#### Field Experiment Using Potable Heat Chambers

The same pair of contrasting genotypes was also subsequently exposed to a short period of elevated temperature (4 days) at anthesis under field conditions using in-field temperature-controlled chambers. The field chamber (FC) experiment was sown in 2016 with two replications per genotype per treatment, adjacent to the larger normally sown field trial of 196 entries. The experiment was sown as a discrete trial for ease of access and chamber installation/monitoring. Three plots of 6 m × 2 m, comprising six rows per plot of each genotype were sown in each replication. At anthesis (Z61), controlled temperature chambers were placed over a 2 × 2 m area in two of the three plots (Supplementary Figure 2) to impose heat stress for 4 days. The chamber design and construction are described in detail by Thistlethwaite et al. (2020). One of the chambers was maintained at ambient temperature (FCT1), and another heated between 10:00 and 16:00 to maintain +6°C above ambient temperature (FCT2) within each replication per genotype. Outside these hours, FCT2 was maintained at ambient temperature. The source of heating in the chambers was from reverse cycle air conditioning, which enabled accurate control of the temperature in the chambers but prevented heating more than 6°C above the ambient. Nevertheless, these maximum temperatures were similar to the high temperatures experienced at Narrabri during a heatwave.

### Characterization of Germplasm

#### Phenology

For glasshouse and chamber experiments, the flag leaf and spikes of the main stems of individual plants were tagged for the physiological trait assessment. Each growth stage was defined using the Zadoks' scale of cereal development (Zadoks et al., 1974). Phenology for each plant was recorded through daily observations. The heading stage was determined when 80% of the inflorescences emerged from the flag leaf sheath (Z57). Anthesis was determined when the first anthers were visible from the middle spikelets of primary tillers (Z61). Physiological maturity was estimated when spikes and most of the peduncle had turned yellow (Z91). The grain filling period was determined as the duration between anthesis and physiological maturity.

#### Field Experiment

The data of the large TOS field experiments were collected following the standard protocols given by Pask et al. (2012) and in detail by Ullah et al. (2020b).

#### Glasshouse Experiment

In the glasshouse experiment, leaf net photosynthesis and transpiration rates of the tagged flag leaves were measured from the start of treatment and then every 5th day until leaves turned yellow in all treatments. Data were recorded using a portable photosynthesis system (LI-6400, LI-COR^®^, Lincoln, NE, USA) with a standard 2 × 3 cm leaf chamber, leaf thermocouple, and a blue–red LED light source. The instrument was calibrated each day before taking measurements. CO_2_ concentration of the inlet air stream was fixed at 400 μmol m^−2^ s^−1^, flow rate 500 μmol s^−1^, block temperature 20–30°C, and photosynthetic photon flux density (PPFD) 1,000 μmol m^−2^ s^−1^.

Chlorophyll content of the tagged flag leaf on the main stems was measured using a portable chlorophyll meter (SPAD 502 Plus, Konica Minolta Sensing, Inc., Osaka, Japan). Data were recorded before starting treatment, at heading, anthesis, and then every 4th day following anthesis until physiological maturity.

Flag leaf temperatures of the main stem were measured every 4th day after starting treatments until the leaves turned yellow using a thermal camera (Ti20 Thermal Imager, Fluke, Everett, WA, USA). Every time, three images were taken per flag leaf per plant and averaged. To determine the flag leaf temperature, these images were processed using the software “Inside IR 4.0” downloaded from: http://www.fluke.com/fluke/auen/infrared-cameras/fluke-ti20.htm?pid=56180.

Leaf area was measured in all treatments in the glasshouse using a leaf area meter (model 3100; LI-COR, Inc., Lincoln, NE, USA) just prior to treatment and then every 6th day until there were no green leaves.

Measurements of individual grain dry weight (IGDW) were made every 6th day from anthesis until physiological maturity. Spikes on the main stem were dissected, and grains from spikelets 8, 9, and 10 were weighed before and after oven drying for 48 h at 70°C according to Dias de Oliveira et al. (2013). Similar procedures were followed for main spike dry weight (MSDW) over time under glasshouse conditions, where spikes were collected from the main stem every 6th day and oven-dried to obtain total dry weight.

Above-ground biomass at anthesis and maturity, plant height, number of tillers per plant, spike length, number of grains per main spike, grain weight per main spike, grains per plant, 1,000 kernel weight (TKW), harvest index, and grain yield were determined using standard protocols (Reynolds et al., 2007). Plants were harvested at maturity and separated into stems and spikes, oven-dried at 70°C for 48 h, and weighed. Spikes were counted and threshed by hand and grain re-dried to constant weight to determine grain yield.

#### Field Chambers

Leaf net photosynthesis and transpiration rates of the tagged flag leaves were measured from the start of treatment and then every 10th day until leaves turned yellow in all treatments. SPAD and NDVI were assessed every 8th day from treatment initiation to maturity. Measurements of IGDW were made every 6th day from anthesis until physiological maturity.

Infrared thermometry systems (IRTs) provided by the CSIRO Plant Phenomics facility Canberra were used for the evaluation of canopy temperature from booting to physiological maturity. IRTs were Smartcrop Automated crop stress monitoring system (Smartfield, Inc., Lubbock, TX, USA) incorporated with Zytemp model TN901 IR thermometer (Zytemp, Hsinchu, Taiwan, ROC). IRTs were wireless and transmitted readings to the controller system (base) connected through a radio link (Mahan and Yeater, 2008; Mahan et al., 2010; Conaty, 2011). A weatherproof data logger (HOBO^®^ U23, Pro v2) was used to measure the temperature and humidity of the trial area where IRTs were used.

Above-ground biomass at anthesis and maturity, plant height, number of tillers, spike length, number of grains per main spike, MSDW, TKW, harvest index, number of grains m^−2^, screening percentages, protein content, and grain yield were determined using standard protocols (Reynolds et al., 2007). A plot of 4 m^2^ exposed to the treatment was harvested manually at maturity and threshed by machine (Kingaroy Engineering, Pty Ltd., Kingaroy, Australia) to obtain grain yield per plot. The yield was then converted to t ha^−1^.

### Statistical Analysis

A residual maximum likelihood (REML) model was fitted using the REML function in GenStat, version 14 (Payne, 2011), and the significance of variance components was estimated for each trait. Genotypes and treatments were considered as fixed terms, and replications within treatments were considered as random terms in the model. Genotype and Genotype × Environment interaction (GGE) biplots of the relationships between genotypes and environments were constructed using the same software, and a dendrogram was constructed based on genetic distances. GraphPad Prism software version 8.2 (GraphPad Software, Inc., San Diego, CA, USA) was used for linear and non-linear regression analyses and to construct figures. Curves were fitted using the least squares fit and Akaike's information criterion (H J Motulsky, Comparison Method, GraphPad Curve Fitting Guide. Accessed October 4, 2019. https://www.graphpad.com/guides/prism/8/curve-fitting/REG_Comparing_Models_Tab.htm). In addition to the sigmoid functions (variate slopes) and dissociation curves described below, quadratic and, for some models, cubic components were fitted. The best-fit model accounted for the greatest percentage of variance.

A logistic growth curve was fitted to MSDW and IGDW (Equation 1):


$$\begin{array}{l} Y=\frac{Y_{\max}.Y_{0}}{\left(\left(Y_{\max}.Y_{0}\right)\exp\left(-kX\right)+Y_{0}\right)} \end{array}$$

where *Y*_max_ is the maximum *y* value, *Y*_0_ is the starting *y* value, and *k* is the rate constant. An asymmetric Gompertz curve (https://www.statforbiology.com/nonlinearregression/Usefulequations) was fitted to SPAD in the glasshouse experiment (Equation 2):


$$\begin{array}{l} Y=c+\left(d-c\right)\left\{1-exp\left\{-exp\left[b\left(X-e\right)\right]\right\}\right\} \end{array}$$

where *b* is the slope around the inflection point, *c* is the lower asymptote, *d* is the higher asymptote, and *e* is the mid-way point (in time) between *c* and *d*.

One-phase dissociation curves were fitted to SPAD values and NDVI in the FC experiment (Equation 3):


$$\begin{array}{l} Y=Plateau-\left(Plateau-Y_{0}\right)\cdot \left(1-exp\;\left(kX\right)\right) \end{array}$$

Thermal time (accumulated daily average temperature, base temperature 3°C for reproductive, and grain filling stage) was calculated from anthesis. R software version 3.1.1 (R Core Team, 2013) was used to plot the data obtained using data loggers (temperature and humidity) and IRTs (canopy temperature).

## Results

### Environmental Fluctuations

Temperature fluctuations for each TOS experiment are provided in Supplementary Figure 1, and further details are given in Ullah et al. (2020b), where late sown experiments (TOS2) experienced higher temperature conditions from flowering onward. The temperature and humidity inside the heat chambers, both FCT1 (ambient) and FCT2 (heated), during the 4 days of treatment are given in Supplementary Figure 3, where plants under FCT2 experienced higher temperature conditions.

### Selection of Contrasting Lines From Large-Scale Field Phenotyping

Anthesis occurred during the first 2 weeks of September in TOS1 and during the 2nd and 3rd week of October in TOS2, depending on the year.

A pair of emmer-derived lines was selected based on their differing response to high temperature (Ullah et al., 2020b), and the results are shown in Table 1. The lines G1 (putative tolerant) and G2 (putative sensitive) had similar yield, yield components, plant height, and phenology under TOS1, but differed in yield under TOS2. Compared with G2 (Table 1), the yield stability of G1 in TOS2 was greater, which was associated with higher NDVI at Z73 (stay-green ability), longer grain filling period, fewer screenings, and greater kernel weight.

**Table 1:** Mean yield, yield components, and growth parameters for two contrasting genotypes for 2015 and 2016 combined field data.

**Fixed term**	**Trt**	**Yield**	**TKW**	**SCR**	**PRO**	**TW**	**DTF**	**GFP**	**PH**	**NDM**	**EGC**
		**(t ha^**−1**^)**	**(g)**	**(%)**	**(%)**	**(kg hL^**−1**^)**	**(days)**	**(days)**	**(cm)**		
G1	TOS1	5.81	51.4	3.10	13.5	80.5	112	52	107.1	0.76	0.29
	TOS2	4.33	39.7	5.02	13.1	78.6	82	39	87.2	0.66	0.25
G2	TOS1	5.72	52.2	3.41	12.9	81.0	113	48	106.2	0.75	0.28
	TOS2	3.02	35.1	7.63	13.8	78.0	81	34	85.3	0.54	0.22
*P*-value	G	*P < * 0.001	*P < * 0.001	*P < * 0.007	*P < * 0.001	*P < * 0.001	*P < * 0.001	*P < * 0.001	*P < * 0.001	*P < * 0.013	*P < * 0.009
	T	*P < * 0.002	*P < * 0.041	*P < * 0.037	NS	NS	*P < * 0.001	*P < * 0.002	*P < * 0.043	*P < * 0.021	NS
	GxT	*P < * 0.001	*P < * 0.001	*P < * 0.049	NS	NS	NS	*P < * 0.049	*P < * 0.001	*P < * 0.049	NS
LSD	G	0.34	2.32	2.01	0.60	1.25	1.42	1.69	2.75	0.04	0.04
	T	0.93	9.87	4.01	-	-	5.37	8.74	6.87	0.13	-
	GxT	0.97	9.95	4.51	-	-	-	8.80	7.01	0.14	-

*The contrasting genotypes were selected from a large-scale screening trial in field conditions under optimum sowing (TOS1) and late sowing (TOS2—heat stressed) for 2 consecutive years and selected based on yield stability. Levels of significance and least significant differences (LSD) are indicated at p < 0.05 according to Tukey post-hoc tests*.

*TKW, 1,000 kernel weight; SCR, screenings; PRO, protein content; TW, test weight; DTF, days to flowering; GFP, grain filling period; PH, plant height; NDM, NDVI at milk stage (Z73); EGC, early ground cover; G, genotype; T, treatment*.

Seed quality and yield-related traits for G1 (encircled blue), G2 (encircled black), and their recurrent hexaploid parent (PBW 502), and the other progenies derived from this family are shown in Supplementary Figure 4. The tolerant line G1 showed phenotypic superiority for grain yield stability, kernel weight, and screenings under stress compared with G2 and the recurrent hexaploid parent.

### Phenology

#### Glasshouse

A non-significant difference (*p* = 0.17) between the two genotypes was observed for days to anthesis in the glasshouse (~79 days). However, there was a significant G × T interaction for the length of the grain-filling period (Table 2). The grain filling period of G1 was reduced by 8 days under CET2 and 5 days under CET3, whereas the grain filling period of G2 was reduced by an extra day under both CET2 and CET3.

**Table 2:** Phenological, morphological, yield, and yield component traits of the two wheat genotypes grown in a glasshouse under ambient conditions (CET1) or exposed to high temperature from heading (CET2) or anthesis (CET3) until maturity.

**Fixed term**	**Trt**	**Yield (g plant^**−1**^)**	**TKW (g plant^**−1**^)**	**HI (%)**	**TNG (plant^**−1**^)**	**MSGN (spike^**−1**^)**	**MSGW (g)**	**AGDB (g plant^**−1**^)**	**PH (cm)**	**GFP (days)**
G1	CET1	19.40	57.40	56.41	333.52	64	3.8	53.61	88.8	42
	CET2	12.58	46.69	50.62	269.54	57	3.0	37.54	84.8	34
	CET3	14.03	48.96	51.61	286.51	58	3.2	41.21	87.3	37
G2	CET1	17.79	54.30	53.81	320.53	60	3.7	50.87	89.0	39
	CET2	8.88	42.09	44.72	211.02	48	2.2	28.74	81.5	30
	CET3	10.06	42.72	46.52	235.33	50	2.4	31.69	86.5	33
*P*-value	G	*P < * 0.001	*P < * 0.001	*P < * 0.004	*P < * 0.001	*P < * 0.001	*P < * 0.001	*P < * 0.001	NS	*P < * 0.001
	T	*P < * 0.001	*P < * 0.001	*P < * 0.001	*P < * 0.001	*P < * 0.001	*P < * 0.001	*P < * 0.001	*P < * 0.044	*P < * 0.001
	GxT	*P < * 0.001	*P < * 0.033	NS	*P < * 0.002	*P < * 0.034	*P < * 0.001	*P < * 0.005	NS	*P < * 0.001
LSD	G	0.44	0.81	1.61	9.69	2.22	0.11	1.38	-	0.23
	T	0.54	1.00	1.97	11.87	2.72	0.13	1.69	1.78	0.29
	GxT	0.76	1.41	-	16.78	3.84	0.19	2.39	-	0.41

*The genotypes were selected for their contrasting yield response under late sowing in the field (G1, putative heat tolerant and G2, putative heat susceptible). Levels of significance and LSD are indicated at p < 0.05 according to Tukey's post-hoc tests*.

*TKW, 1,000 kernel weight; HI, harvest index; TNG, total number of grains per plant; MSGN, number of grains per main spike; MSGW, main spike grain weight; AGDB, above-ground dry biomass at maturity; PH, plant height; GFP, grain filling period; G, genotype; T, treatment; NS, Non-significant*.

#### Field Chambers

G1 and G2 flowered at the same time (109 days) prior to the imposition of heat treatments in the FCs (*p* = 0.49). However, a G × T interaction was observed for the duration of the grain-filling period after the imposition of FCT2 (Table 3). Under FCT1, the grain filling period of G2 was 4 days shorter than G1. However, the duration was reduced by 7 days in G2 and 4 days in G1 under FCT2.

**Table 3:** Phenology, quality, yield, and yield components of the two wheat genotypes selected for their contrasting yield response under late sowing in the field (G1, putative heat tolerant and G2, putative heat susceptible).

**Fixed term**		**^**1**^Yield (t ha^**−1**^)**	**TKW (g)**	**HI (%)**	**MSGN (spike^**−1**^)**	**MSDW (g)**	**GN (m^**−2**^)**	**SCR (%)**	**PRO (%)**	**AGDB (kg m^**−2**^)**	**ADBA (kg m^**−2**^)**	**PH (cm)**	**SL (cm)**	**GFP (days)**
G1	FCT1	5.73	55.11	56.51	56.17	2.97	9,937	1.95	13.09	2.25	1.58	99.5	13.22	56
	FCT2	5.17	51.10	50.50	48.51	2.34	9,623	2.48	13.44	2.03	1.54	99.1	13.21	52
G2	FCT1	5.24	53.58	52.00	52.33	2.49	9,167	1.86	13.69	2.07	1.43	99.2	13.23	52
	FCT2	4.04	47.40	45.50	39.11	2.04	8,037	3.16	14.55	1.87	1.44	99.1	13.20	45
*P*-value	G	*P < * 0.001	*P < * 0.001	*P < * 0.001	*P < * 0.001	*P < * 0.001	*P < * 0.001	*P < * 0.023	*P < * 0.003	*P < * 0.034	*P < * 0.029	NS	NS	*P < * 0.001
	T	*P < * 0.001	*P < * 0.001	*P < * 0.001	*P < * 0.001	*P < * 0.001	*P < * 0.001	*P < * 0.001	*P < * 0.012	*P < * 0.007	-	NS	NS	*P < * 0.001
	GxT	*P < * 0.039	*P < * 0.041	NS	*P < * 0.035	*P < * 0.031	*P < * 0.019	*P < * 0.008	NS	NS	-	NS	NS	*P < * 0.025
LSD	G	0.23	1.75	0.98	1.01	0.11	350	0.21	0.36	192	0.07	-	-	0.98
	T	0.27	1.97	1.07	1.23	0.14	473	0.25	0.41	203	-	-	-	1.27
	GxT	0.33	2.01	-	3.99	0.21	607	0.30	-	-	-	-	-	1.38

*Genotypes were grown in field conditions exposed to ambient chamber at anthesis (FCT1) or heated chambers at anthesis (FCT2) for 4 consecutive days. Levels of significance and LSD are indicated at p < 0.05 according to Tukey post-hoc tests*.

*TKW, 1,000 kernel weight; HI, harvest index; MSGN, number of grains per main spike; MSDW, dry weight per main spike; GN, number of grains per m^2^; SCR, screenings; PRO, protein content; AGDB, above-ground dry biomass at maturity; ADBA, above-ground dry biomass at anthesis; PH, plant height; SL, spike length; GFP, grain filling period; T, treatment; G, genotype; NS, Non-significant*.

### Plant Morphological and Agronomical Traits

#### Glasshouse

Above-ground dry matter production and height of G1 and G2 were not significantly different in the glasshouse before the imposition of heat treatment, but CET2 treatment reduced the plant height of both genotypes (Table 2). Significant G × T interactions were observed for yield and yield components in the glasshouse conditions. Although both genotypes yielded more under CET1 conditions (followed by CET3 and then CET2), and G1 always yielded more than G2, the reduction in yield due to the heat treatments was greater for G2 than G1. The grain yield of G1 was 35% lower under CET2 and 28% lower under CET3 than under CET1. In contrast, the grain yield of G2 was reduced similarly under CET2 and CET3 by about 50% compared with CET1.

G × T interactions were observed for the number of grains on the main spike, total grain number per plant, main spike grain weight, TKW, and above-ground biomass (Table 2). Under CET1, both genotypes had similar number of grains on the main spike, main spike grain weight, total number of grains per plant, and above-ground biomass; however, G1 had greater TKW. The reductions in trait values were always more severe for G2 than G1 under CET2 and CET3, except for the total grain number per plant. For the harvest index, only the main effects were significant and G1 had a higher harvest index.

**Figure 1:**
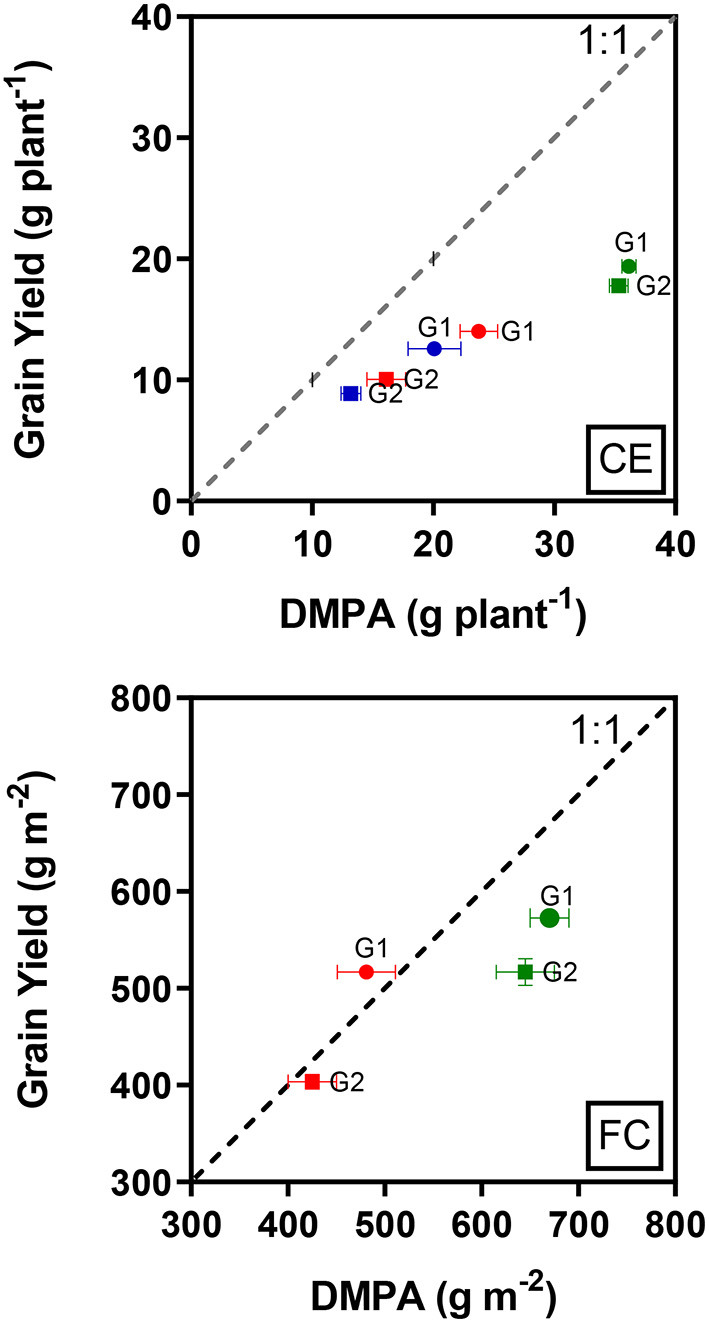
Relationship between mean grain yield and dry biomass produced after anthesis (DMPA) in the two wheat genotypes (G1, putative heat tolerant and G2, putative heat susceptible) in response to day/night temperatures of CET1 [control (22/14°C), green symbols], CET2 (30/20°C from heading, blue symbols), and CET3 (30/20°C from anthesis, red symbols) under controlled environment conditions (CE) or field chamber (FC) conditions (FCT1, ambient temperature chamber, green symbols; FCT2, heated chamber, red symbols). The dashed line represents 1:1 and error bars represent ± 1 SEM.

The total above-ground biomass produced after anthesis was not fully converted into grain yield under all treatments (Figure 1, CE), but CET3 followed by CET2 induced better conversion rates than CET1. However, although G2 produced less biomass after anthesis compared to G1, G2 converted biomass into yield more effectively.

#### Field Chambers

Above-ground biomass was higher in G1 than G2 prior to the imposition of heat stress in the field (Table 3). Plant height and spike length at maturity were not affected by genotypes or treatments. Only genotypes and the main effects of the treatment were significant for the above-ground biomass at maturity, harvest index, and grain protein content. Greater dry biomass at maturity and higher harvest index were observed in G1; however, G2 produced higher grain protein content. Significant GxT interactions were observed for grain yield, TKW, number of grains m^−2^, main spike grain number and weight, and screening percentage. Heat treatment induced a greater reduction in these traits in G2 than G1.

In the heat chamber experiment under ambient conditions, both genotypes did not fully convert their total above-ground biomass produced after anthesis to grain yield. However, although the biomass produced after anthesis was reduced by the heat treatment, G1 produced the grain yield that is equal to the biomass production (Figure 1, FC).

### Spike Weight and Grain Growth Rates

#### Glasshouse

A significant G × T interaction (*p* < 0.037) was observed for MSDW in the glasshouse. The change in MSDW with thermal time after anthesis was best fitted by logistic growth curves (Equation 1), which explained 91–96% of the observed variation. The starting MSDW was constrained to the mean of the data at time 0 (0.89 g, i.e., preheat treatments) as there was no significant difference between the groups (*p* > 0.05). The remaining curve parameters differed between the data sets (Supplementary Figure 5 and Supplementary Table 2). In general, the heat stress increased the initial rate of dry weight accumulation in the main spikes but reduced the growing degree days when the rate of accumulation slowed down compared with control conditions, especially when the heat stress was applied from heading. Under CET2 and CET3, MSDW initially increased at similar rates per degree day for both genotypes, but the rate of accumulation began to slow down earlier in G2 compared with G1, as indicated by the inflection points of the curves.

**Figure 2:**
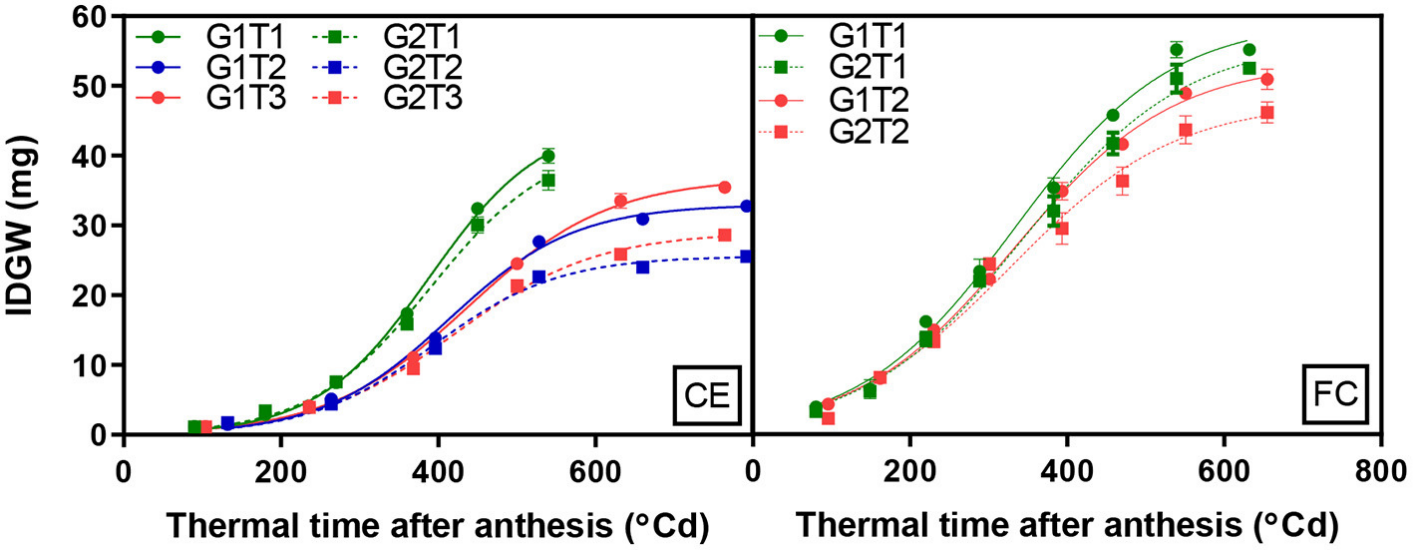
Change in individual grain dry weight (IGDW) of grains from spikelets 8, 9, and 10 on the main spike over time for two wheat genotypes (G1, putative heat tolerant, circles and solid lines; G2, putative heat susceptible, squares and dotted lines) in response to day/night temperatures of CET1 [control (22/14°C)], CET2 (30/20°C from heading), and CET3 (30/20°C from anthesis) under controlled environment conditions (CE) or heat chambers in the field (FC) (FCT1, ambient temperature chamber and FCT2, heated chamber) on IGDW. Error bars represent SEM, *n* = 4.

A significant G × T interaction was observed for IGDW (*p* < 0.001). IGDW accumulation after anthesis was best fitted by logistic growth curves (Equation 1), which explained 99% of the variation (Figure 2, CE and Table 4). IGDW started to increase after slightly less thermal time and at a slightly higher rate in G1 compared to G2 under control conditions. However, the lag phase in grain growth was longer under CET2 and CET3 where more thermal time was required before grain weight started to increase (see Xint in Table 4). Grain weight also accumulated at slower rates under heat stress with the effect more detrimental to G2 whose final grain mass was lower. Interestingly, although IGDW of G2 was impacted more by high temperature, the heat treatment applied from heading (CET2) or anthesis (CET3) on each genotype imposed similar restrictions on grain growth until quite late in development (>500°Cd) when IGDW was inhibited more by CET2 than CET3.

**Table 4:** Parameters of the logistic curves fitted to IGDW over time in Figure 2 for two wheat genotypes (G1, putative heat tolerant and G2, putative heat susceptible) in response to day/night temperatures of CET1 [control (22/14°C)], CET2 (30/20°C from heading), and CET3 (30/20°C from anthesis) under glasshouse conditions (CE) or FC conditions (FCT1, ambient temperature chamber and FCT2, heated chamber).

	**GxT**	**Y_**max**_ (mg)**	**Y_**0**_ (mg)**	**k (^**°**^Cd^**−1**^ x 10^**−3**^)**	**Xint (^**°**^Cd)**	***R* ^**2**^**
CE	G1CET1	45.26	0.20	13.87	72.11	0.99
	G2CET1	42.24	0.27	12.96	77.14	0.99
	G1CET2	33.00	0.19	12.48	80.14	0.99
	G2CET2	25.61	0.18	12.59	79.43	0.99
	G1CET3	36.81	0.28	11.15	89.72	0.99
	G2CET3	29.04	0.26	11.15	89.72	0.99
FC	G1FCT1	59.33	1.97	10.11	98.95	0.99
	G2FCT1	56.58	1.87	9.76	102.4	0.99
	G1FCT2	53.39	1.91	9.92	100.9	0.99
	G2FCT2	47.77	2.05	9.49	105.4	0.98

*Where Y_max_ is maximum IGDW, Y_0_ is starting IGDW at anthesis, k is the rate constant, and Xint is the time when IGDW starts to increase. G × T, genotype and treatment. Best fit curves for each genotype treatment combination were significantly different from each other (p < 0.01)*.

#### Field Chambers

A significant G × T interaction was observed for IGDW (*p* < 0.001). The logistic curve fit to the data explained 97% of the variance for this trait (Figure 2, FC and Table 4). Under short periods of heat stress induced by heat chambers in the field, genotypes needed more thermal time to reach their maximum dry weight due to slower grain mass accumulation. After a short lag phase, the grain growth rate increased rapidly with FCT2 increasing at a slower rate compared with FCT1. In this scenario, G1 had faster grain growth rates under both treatments and produced the highest IGDW. Average IGDW accumulation over time under heat stress was restricted by 7% in G1 and 13% in G2 under FCT2 conditions.

In comparing the FC treatments with the glasshouse on IGDW, there was a shorter lag phase in the heat chamber responses, the initial growth rates, which are proportional to *y*(1 – *y*/*yM*), were higher and the final grain weight was larger.

### Stay-Green Traits

#### Glasshouse

**Figure 3:**
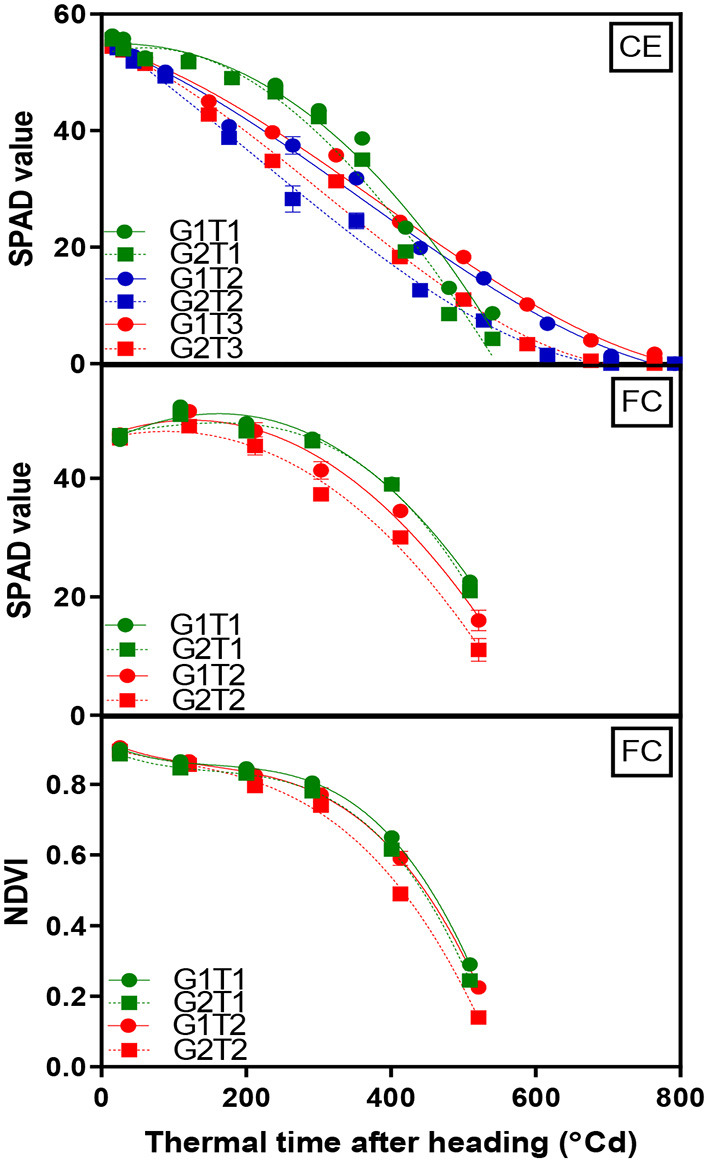
Change in mean chlorophyll content (SPAD) of the flag leaf and canopy greenness (NDVI) over time for two wheat genotypes (G1, putative heat tolerant, circles and solid lines; G2, putative heat susceptible, squares and dotted lines) in response to day/night temperatures of 22/14°C (control, CET1), 30/20°C from heading (CET2), and 30/20°C from anthesis (CET3) under controlled environment conditions (CE) or heat chambers in the field (FC) (FCT1, ambient temperature chamber and FCT2, heated chamber). Error bars represent SEM, *n* = 4.

A significant G × T interaction (*p* < 0.001) for chlorophyll content as indicated by SPAD readings was observed in the glasshouse where heat stress increased senescence rates in both genotypes. The decline in chlorophyll content over time was fitted using Equation (2) with the lower asymptote constrained to 0 and the fitted curves accounted for ~98% of the variation (Figure 3, CE and Supplementary Table 3). Under optimum conditions, both genotypes behaved similarly where chlorophyll content did not initially decline until around 300°Cd. Under heat stress, chlorophyll content decreased much earlier, particularly when the heat treatment was applied from the heading stage and more rapidly in G2 with the mid-way point for the decline occurring at 132°Cd earlier compared with 70°Cd earlier for G1.

Genotypes and the main effects of the treatment were significant (*p* < 0.001) for the green leaf area over time; however, no significant G × T interaction was observed. G1 exhibited a larger whole plant green leaf area and high temperature diminished the green leaf area in both genotypes (Supplementary Figure 6 and Supplementary Table 4).

#### Field Chambers

A significant G × T interaction (*p* < 0.001) for chlorophyll content was observed in the heat chamber experiment. Data were fitted using a dissociation function (Equation 3), which accounted for 98% of the variation (Figure 3, FC). The heat stress treatment accelerated the rate of the loss of flag leaf chlorophyll content and was more rapid in G2 compared to G1. The same function (Equation 3) was fitted to the NDVI data, which accounted for 99% of the variation (Figure 3, FC). Genotypes and the main effects of the treatment were highly significant (*p* < 0.001) for NDVI over time; however, a G × T interaction was non-significant. G1 exhibited greater NDVI at grain filling in both treatments.

### Gas Exchange Parameters

#### Glasshouse

**Figure 4:**
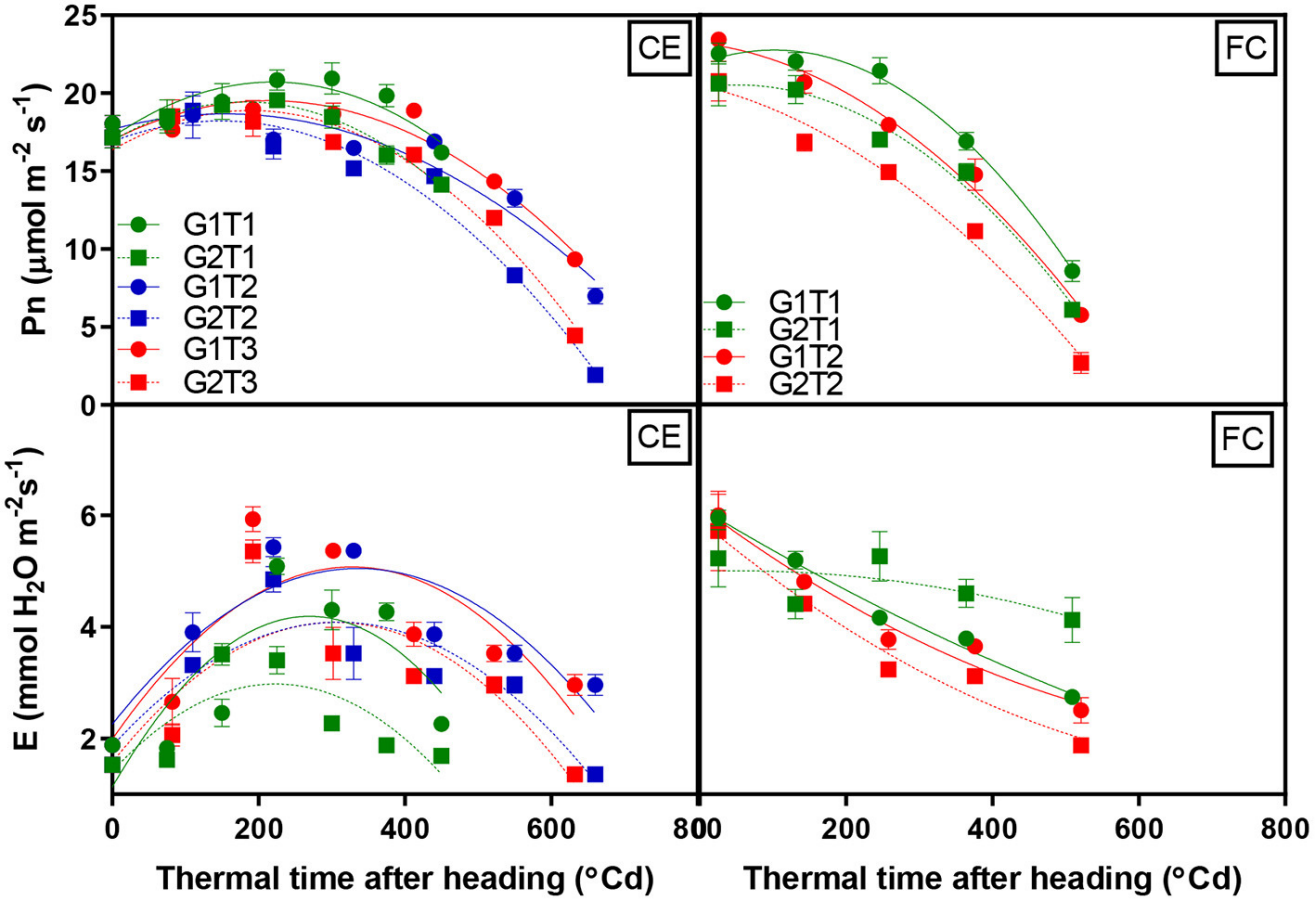
Change in net photosynthesis and transpiration rates over time for two wheat genotypes (G1, putative heat tolerant, circles and solid lines; G2, putative heat susceptible, squares and dotted lines) in response to day/night temperatures of CET1 [control (22/14°C)], CET2 (30/20°C from the heading), and CET3 (30/20°C from anthesis) under controlled environment conditions (CE) or heat chambers in the field (FC) (FCT1, ambient temperature chamber and FCT2, heated chamber). Error bars represent SEM, *n* = 4.

A significant G × T interaction (*p* < 0.047) was observed for the leaf net photosynthetic rate (Pn) in the glasshouse, which was fitted by a second-order polynomial regression, including the quadratic terms that described 89% of the variation (Figure 4, CE). Pn was similar before imposing heat treatment; however, high-temperature stress reduced photosynthetic activity in both genotypes. Pn started to decline after 10 days in the high-temperature treatments, and G2 declined more rapidly than G1. There was a significant G × T interaction observed for the leaf transpiration rate (*p* < 0.041). Under heat stress, transpiration rates (E) were higher compared to CET1. Transpiration rates were fitted by a second-order polynomial regression, including the quadratic terms that accounted for 77% of the variation (Figure 4, CE). G1 had greater leaf transpiration than G2 in both control- and heat-stressed environments.

#### Field Chambers

A significant G × T interaction (*p* < 0.043) was observed for Pn in the heat chamber experiment. Pn over time was fitted by a second-order polynomial regression, including quadratic terms that described 93% of the variation (Figure 4, FC). Genotypes exhibited different trends in Pn almost 1 week after treatment, and the greatest reduction was observed for G2 in FCT2. While a G × T interaction for leaf transpiration was non-significant, genotypes and the main effects of the treatment were significant (*p* < 0.001). Overall, G1 showed a greater average transpirational rate. Leaf transpiration rates over time were fitted by a second-order polynomial regression, including quadratic terms and 93% of the variation in this trait was explained (Figure 4, FC).

**Figure 5:**
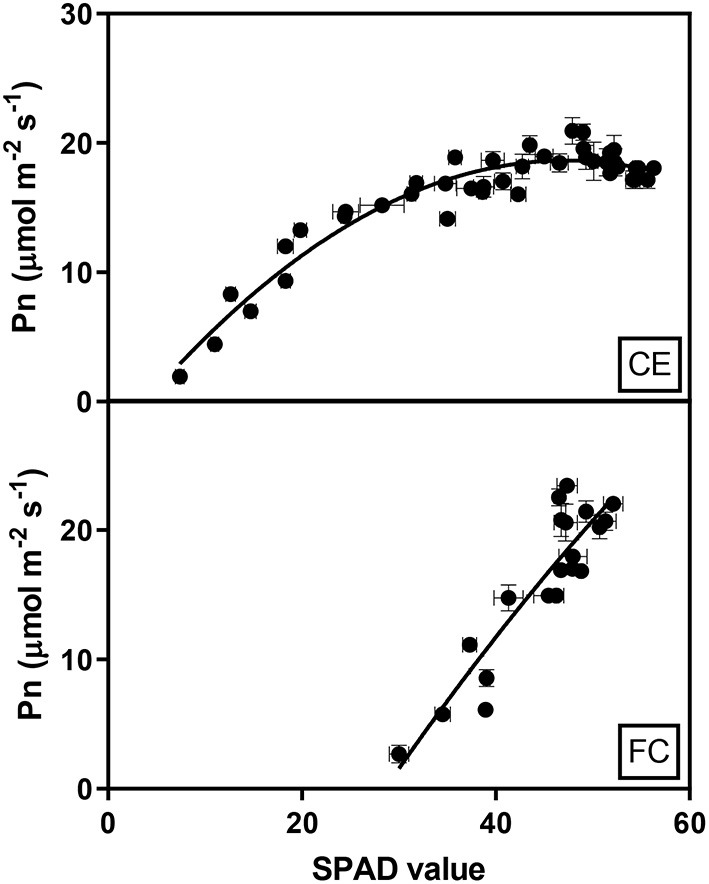
Relationship between the mean net photosynthesis (Pn) and chlorophyll contents (SPAD) of flag leaves in two wheat genotypes (G1, putative heat tolerant and G2, putative heat susceptible) under controlled environment conditions (CE) or heat chambers in the field (FC). Data were combined within an environment due to no significant difference between temperature treatments and genotypes (*p* > 0.05). Error bars represent SEM. The equation fit to the CE data is Pn = −3.38+0.93SPAD-0.01SPAD^2^ and the equation for FC data is Pn = 0.94SPAD-26.02.

The relationship between chlorophyll content and Pn reduction over time for the glasshouse experiment was fitted by a second-order polynomial regression, including quadratic terms, which described 85% of the variation and demonstrated that Pn increased rapidly with increasing chlorophyll content, which plateaued around 40 SPAD units. A simple linear regression (*R*^2^ 0.81) described the relationship between Pn and chlorophyll content in the heat chamber experiment where photosynthesis initially occurred at higher SPAD units than in the glasshouse and increased by 0.94 μmol m^−2^ s^−1^ for each SPAD unit as indicated by the slope of the regression (Figure 5).

### Canopy Temperature

#### Glasshouse

A G × T interaction for the flag leaf temperature was non-significant in the glasshouse; however, genotypes and main effects of the treatment were significant (*p* < 0.001). Compared to the control environment, the flag leaf temperature was observed to be higher under heat stress, and G2 had a comparatively warmer leaf temperature in all treatments (data not shown).

#### Field Chambers

Changes in canopy temperature in the field occurred following the short period of heat shock. G2 had a comparatively warmer canopy at early grain filling and increased with the growth stage. However, G1 maintained a relatively cooler canopy during and following heat shock (Supplementary Figure 7).

## Discussion

Little information is available on the use of emmer wheat to improve the heat tolerance of bread wheat through direct crossing, but the potential of emmer wheat as a source of new allelic variation for heat tolerance has already been discussed in our previous work (Ullah et al., 2021a,b). This article focuses on the response of two related emmer-derived lines to high temperature and showed that the tolerance to high temperature was associated with different mechanisms, controlled by genes transferred from emmer wheat. Both emmer-derived lines had the same hexaploid recurrent parent but their emmer parents differed. G1 was more stable across different environmental conditions. It is likely that the emmer parent of G1 had superior heat stress tolerance to that of G2, given their contrasting response to heat. However, it is difficult to determine with certainty as the emmer parents were not included in the field experiments because of their poor agronomic characteristics. The pair of emmer derived lines evaluated formed part of a larger marker-trait association analysis published previously (Ullah et al., 2021b). However, it was difficult to determine, categorically, the contribution of emmer to heat tolerance as emmer-specific molecular markers could not be identified from the 90K SNP bread wheat assay. Instead, the emmer parents used to make the derived lines were selected based on their molecular diversity and contrasting performance under heat and drought stress in different environments (Zaharieva et al., 2010).

The use of a combination of phenotyping strategies was effective in identifying and confirming heat stress response in this study. Several traits were identified that were related to the heat-tolerance phenotype of higher yields under field conditions. However, their robustness or repeatability was unconfirmed. For this reason, CE evaluation was used to confirm the previous field responses of the selected materials and to determine how these traits contributed heat tolerance. It is difficult to compare glasshouse conditions with the field as root constraints in pot experiments can impose an obstacle to accurate phenotyping. In addition, the results of TOS experiments in the field may be an artifact of abnormal biomass development in late sown materials and may not be indicative of true heat tolerance. Hence, portable in-field temperature-controlled chambers were used to validate the results of TOS experiments on normally sown materials and to confirm glasshouse observations. Trait expression was more severely reduced following extended heat stress in the glasshouse compared with short periods of heat shock in the FCs. Nevertheless, the rank of genotypes was relatively similar across different environmental conditions, thus making both strategies effective for germplasm selection and breeding. A relatively strong relationship between the performance of the selected genotypes in the three methods (delayed sowing, glasshouse, and field-based heat chambers) indicated that a large-scale screening, using TOS, could be effective for identifying heat-tolerant germplasm as suggested by Telfer et al. (2018).

Heat stress (≥30°C) at early reproductive and grain filling stages had an adverse impact on grain yield through a reduction in the number of grains per spike and grain weight. Average grain yield was reduced by 39% under extended heat stress and by 16% following short periods of high-temperature stress. Similar results for temperatures ≥30°C at the reproductive stage have been reported in wheat following the exposure to variable heat stress (Talukder et al., 2014; Cossani and Reynolds, 2015; Dreccer et al., 2018).

There is often a trade-off between increasing grain number and a reduction in the grain size (Alonso et al., 2018; Quintero et al., 2018); however, G1 produced both greater grain number and size under heat stress, indicating that it may be possible to produce wheat genotypes optimized for both traits (Fahy et al., 2018). For this instance, an improvement for one trait by breeding causing a trade-off effect on another should not be confused. Ji et al. (2010) reported that, under stress, variation in grain number and size was partially governed by different independent genetic regions, suggesting that genetic optimization is possible.

Terminal heat stress reduced above-ground dry biomass in both genotypes compared to their respective control treatments, and G × T interactions were significant only when plants were exposed to an extended period of heat stress. Thus, the extended period of heat stress is likely to be more damaging compared to shorter periods (albeit depending on the temperature reached and timing), but a significant interaction indicates that genetic improvement should be possible. Overall, the higher biomass and yield of G1 relate to its ability to stay-green for longer with slower leaf senescence as observed previously (Pradhan et al., 2012; Cossani and Reynolds, 2015; Liu et al., 2016). Heat-tolerant emmer-derived synthetic wheat lines were also shown to produce greater biomass at anthesis and maturity under warm temperatures (Pinto et al., 2017); however, their stay-green character was not classified.

The emmer-based heat-tolerant line G1 had greater yield stability and was linked to better “stay-green capacity.” This was confirmed under controlled and field heat chamber conditions, where stay-green supported photosynthetic capacity as demonstrated by the relationship between flag leaf chlorophyll content and photosynthesis. These findings indicate that current photosynthesis is important for maintaining grain filling under heat stress in addition to biomass conversion.

Genotypes had a comparatively longer grain filling period in the chamber experiment compared with the glasshouse, which likely reflects the different intensities of the applied stresses. G1 had greater chlorophyll content and a longer grain filling period under heat stress conditions. This positive association under higher temperature can be referred to as the potential of stay-green genotypes to supply assimilates for grain filling processes (Kumari et al., 2013; Shirdelmoghanloo et al., 2016a). The superior stay-green nature of G1 and its higher chlorophyll content enabled higher photosynthesis and subsequent carbon allocation to developing grains. Thus, the reduction of photosynthesis in G2 is likely a function of the rapid leaf senescence and chlorophyll content, leading to a shorter grain filling period. The ability to maintain photosynthesis for yield stability has been shown to be important in other abiotic stress studies (Talukder et al., 2013; Dias de Oliveira et al., 2015).

The higher transpiration rate of G1 under heat stress was observed similar to the photosynthetic response, and a parallel reduction observed at later grain fill suggested a commensurate reduction in stomatal conductance. A higher transpiration rate suggests a greater potential water use, which would be disadvantageous in many wheat growing regions that experience terminal heat and drought stress. Albeit the transpiration measurements were conducted at the leaf level and thus might not reflect the whole plant water use.

The lower canopy temperature observed under heat stress in G1 might be associated with a slower deterioration of chlorophyll content and greater transpiration rates. The link between the functionality of stay-green and low canopy temperature under stressful environments has been described by Lopes and Reynolds (2012). Nevertheless, a high G × T interaction and low heritability are major challenges to the selection for optimized canopy temperature (Rebetzke et al., 2013; Deery et al., 2016).

## Conclusion

The contrasting performance of the pair of related emmer-derived lines at high temperature indicated that heat stress tolerance was under genetic control, which should be further studied. Superior grain yield in the heat-tolerant genotype under heat stress was supported by better stability in photosynthetic capacity due to slower leaf senescence and the stay-green trait. This resulted in superior grain filling rates and larger overall grain weight. The results obtained from the glasshouse and in-field temperature chamber conditions demonstrated that the mechanism is relevant for extended or short periods of heat stress. Moreover, the stay-green trait and superior photosynthetic capacity could be introduced from emmer wheat into commercial wheat cultivars to improve crop performance under high temperatures.

## Data Availability Statement

The datasets presented in this study can be found in online repositories. The names of the repository/repositories and accession number(s) can be found in the article/Supplementary Material.

## Author Contributions

SU, HB, and RT designed the experiments. SU collected the data, performed the REML analyses, and wrote the first draft of the article. HB and SU performed the regression analyses and plotted the results. HB and RT aided in the conception and design of the study, analysis and interpretation of the data, obtaining funding, and approved the final version to be submitted. All authors contributed to the article and approved the submitted version.

## Funding

This research was provided by the Grains Research and Development Corporation (US00057, US00080, and US00081) and the Generation Challenge Program.

## Conflict of Interest

The authors declare that the research was conducted in the absence of any commercial or financial relationships that could be construed as a potential conflict of interest.

## Publisher's Note

All claims expressed in this article are solely those of the authors and do not necessarily represent those of their affiliated organizations, or those of the publisher, the editors and the reviewers. Any product that may be evaluated in this article, or claim that may be made by its manufacturer, is not guaranteed or endorsed by the publisher.
