# Supplementary material for: The Physiological Basis of Improved Heat Tolerance in Selected Emmer-Derived Hexaploid Wheat Genotypes
Source: Front Plant Sci. 2021 Oct 7;12:739246. doi: 10.3389/fpls.2021.739246 (PMC8544522; doi:10.3389/fpls.2021.739246)
Supplement: Supplementary file 1 [file Data_Sheet_1.pdf]

Supplementary material

The physiological basis of improved heat tolerance in selected emmer-derived hexaploid wheat genotypes

Smi Ullah[1], Richard Trethowan[1, 2] and Helen Bramley[1]

[1]School of Life and Environmental Sciences, Plant Breeding Institute, Sydney Institute of Agriculture, The University of Sydney, Narrabri 2390, NSW, Australia

[2]School of Life and Environmental Sciences, Plant Breeding Institute, Sydney Institute of Agriculture, University of Sydney, Cobbitty 2570, NSW, Australia

Supplementary Table 1; List of plant material used in field trials during 2015 and 2016.

2015 ID	2016 ID	DESIGNATION	PEDIGREE
1	483	SUNTOP	-
2	485	SPITFIRE	-
3	478	PBW502	-
4	479	PBW550	-
5	476	DBW16	-
6	477	DBW17	-
7	475	BERKUT	-
8	480	SOKOLL	-
9	474	2-49/CUNNINGHAM//KENNEDY	-
10	543	SUNLIN	-
11	487	EGA GREGORY	-
12	8	PBI09C002-BC-DH16	BERKUT/2/BERKUT / 35891 M500281
13	32	PBI09C004-BC-DH63	BERKUT/2/BERKUT / 35883 M500110
14	318	PBI09C048-BC-DH28	DBW17 /2/DBW17 / 21758 KC75
15	80	PBI09C009-BC-DH62	SOKOLL/2/SOKOLL / 35888 M 500132
16	233	PBI09C038-BC-DH3	PBW550 /2/PBW550 / 18293 KC75
17	86	PBI09C009-BC-DH72	SOKOLL/2/SOKOLL / 35888 M 500132
18	241	PBI09C038-BC-DH18	PBW550 /2/PBW550 / 18293 KC75
19	271	PBI09C043-BC-DH26	DBW16 /2/DBW16 / 21758 KC75
20	39	PBI09C004-BC-DH88	BERKUT/2/BERKUT / 35883 M500110
**21**	**227 (G1)**	**PBI09C035-BC-DH39**	**PBW502 /2/PBW502 / 19385 KC75**
22	2	PBI09C001-BC-DH43	BERKUT/2/BERKUT / 35880 M C18644
23	238	PBI09C038-BC-DH13	PBW550 /2/PBW550 / 18293 KC75
24	6	PBI09C002-BC-DH3	BERKUT/2/BERKUT / 35891 M500281
25	79	PBI09C009-BC-DH60	SOKOLL/2/SOKOLL / 35888 M 500132
26	312	PBI09C048-BC-DH16	DBW17 /2/DBW17 / 21758 KC75
27	49	PBI09C004-BC-DH116	BERKUT/2/BERKUT / 35883 M500110
28	188	PBI09C034-BC-DH4	PBW502 /2/PBW502 / 21758 KC75
29	55	PBI09C008-BC-DH5	SOKOLL/2/SOKOLL / 35883 M500110
30	296	PBI09C045-BC-DH16	DBW16 /2/DBW16 / 18341 KC75
31	225	PBI09C035-BC-DH35	PBW502 /2/PBW502 / 19385 KC75
32	262	PBI09C039-BC-DH79	PBW550 /2/PBW550 / 18343 KC75
33	285	PBI09C043-BC-DH62	DBW16 /2/DBW16 / 21758 KC75
34	320	PBI09C048-BC-DH35	DBW17 /2/DBW17 / 21758 KC75
35	57	PBI09C008-BC-DH11	SOKOLL/2/SOKOLL / 35883 M500110
36	317	PBI09C048-BC-DH25	DBW17 /2/DBW17 / 21758 KC75
37	4	PBI09C001-BC-DH70	BERKUT/2/BERKUT / 35880 M C18644
38	308	PBI09C048-BC-DH11	DBW17 /2/DBW17 / 21758 KC75
39	185	PBI09C034-BC-DH1	PBW502 /2/PBW502 / 21758 KC75
40	273	PBI09C043-BC-DH35	DBW16 /2/DBW16 / 21758 KC75
41	44	PBI09C004-BC-DH105	BERKUT/2/BERKUT / 35883 M500110
42	62	PBI09C009-BC-DH13	SOKOLL/2/SOKOLL / 35888 M 500132
43	261	PBI09C039-BC-DH77	PBW550 /2/PBW550 / 18343 KC75
44	52	PBI09C004-BC-DH127	BERKUT/2/BERKUT / 35883 M500110
45	61	PBI09C009-BC-DH12	SOKOLL/2/SOKOLL / 35888 M 500132
46	298	PBI09C045-BC-DH22	DBW16 /2/DBW16 / 18341 KC75
47	224	PBI09C035-BC-DH33	PBW502 /2/PBW502 / 19385 KC75
48	165	PBI09C028-BC-DH21	WAXWING*2/KIRITATI /3/WAXWING*2/KIRITATI /2/ 35880 M C18644
49	306	PBI09C048-BC-DH9	DBW17 /2/DBW17 / 21758 KC75
50	193	PBI09C034-BC-DH11	PBW502 /2/PBW502 / 21758 KC75
51	230	PBI09C035-BC-DH43	PBW502 /2/PBW502 / 19385 KC75
52	226	PBI09C035-BC-DH38	PBW502 /2/PBW502 / 19385 KC75
53	96	PBI09C009-BC-DH94	SOKOLL/2/SOKOLL / 35888 M 500132
54	287	PBI09C043-BC-DH64	DBW16 /2/DBW16 / 21758 KC75

55	275	PBI09C043-BC-DH42	DBW16 /2/DBW16 / 21758 KC75
56	186	PBI09C034-BC-DH2	PBW502 /2/PBW502 / 21758 KC75
57	133	PBI09C026-BC-DH37	WAXWING*2/KIRITATI /3/WAXWING*2/KIRITATI /2/ 35888 M 500132
58	201	PBI09C034-BC-DH37	PBW502 /2/PBW502 / 21758 KC75
59	272	PBI09C043-BC-DH32	DBW16 /2/DBW16 / 21758 KC75
60	20	PBI09C004-BC-DH27	BERKUT/2/BERKUT / 35883 M500110
61	288	PBI09C043-BC-DH66	DBW16 /2/DBW16 / 21758 KC75
62	284	PBI09C043-BC-DH61	DBW16 /2/DBW16 / 21758 KC75
63	156	PBI09C026-BC-DH96	WAXWING*2/KIRITATI /3/WAXWING*2/KIRITATI /2/ 35888 M 500132
64	177	PBI09C028-BC-DH42	WAXWING*2/KIRITATI /3/WAXWING*2/KIRITATI /2/ 35880 M C18644
65	319	PBI09C048-BC-DH31	DBW17 /2/DBW17 / 21758 KC75
66	12	PBI09C003-BC-DH3	BERKUT/2/BERKUT / 35879 M C18643
67	85	PBI09C009-BC-DH70	SOKOLL/2/SOKOLL / 35888 M 500132
68	216	PBI09C035-BC-DH16	PBW502 /2/PBW502 / 19385 KC75
69	294	PBI09C045-BC-DH12	DBW16 /2/DBW16 / 18341 KC75
70	83	PBI09C009-BC-DH67	SOKOLL/2/SOKOLL / 35888 M 500132
71	309	PBI09C048-BC-DH13	DBW17 /2/DBW17 / 21758 KC75
72	88	PBI09C009-BC-DH77	SOKOLL/2/SOKOLL / 35888 M 500132
73	134	PBI09C026-BC-DH41	WAXWING*2/KIRITATI /3/WAXWING*2/KIRITATI /2/ 35888 M 500132
74	295	PBI09C045-BC-DH14	DBW16 /2/DBW16 / 18341 KC75
75	200	PBI09C034-BC-DH36	PBW502 /2/PBW502 / 21758 KC75
76	18	PBI09C004-BC-DH16	BERKUT/2/BERKUT / 35883 M500110
77	58	PBI09C008-BC-DH13	SOKOLL/2/SOKOLL / 35883 M500110
78	169	PBI09C028-BC-DH30	WAXWING*2/KIRITATI /3/WAXWING*2/KIRITATI /2/ 35880 M C18644
79	256	PBI09C039-BC-DH61	PBW550 /2/PBW550 / 18343 KC75
80	113	PBI09C026-BC-DH5	WAXWING*2/KIRITATI /3/WAXWING*2/KIRITATI /2/ 35888 M 500132
81	98	PBI09C009-BC-DH99	SOKOLL/2/SOKOLL / 35888 M 500132
82	40	PBI09C004-BC-DH89	BERKUT/2/BERKUT / 35883 M500110
83	215	PBI09C035-BC-DH15	PBW502 /2/PBW502 / 19385 KC75
84	13	PBI09C003-BC-DH4	BERKUT/2/BERKUT / 35879 M C18643
85	107	PBI09C021-BC-DH9	T.DICOCCONP194625/AE.SQUARROSA(372)/2/3*PASTOR /4/T.DICOCCONP194625/AE.SQUARROSA(372)/2/3*PASTOR /3/ 35883 M500110
86	171	PBI09C028-BC-DH33	WAXWING*2/KIRITATI /3/WAXWING*2/KIRITATI /2/ 35880 M C18644
**87**	**196 (G2)**	**PBI09C034-BC-DH22**	**PBW502 /2/PBW502 / 21758 KC75**
88	229	PBI09C035-BC-DH42	PBW502 /2/PBW502 / 19385 KC75
89	263	PBI09C039-BC-DH83	PBW550 /2/PBW550 / 18343 KC75
90	159	PBI09C026-BC-DH115	WAXWING*2/KIRITATI /3/WAXWING*2/KIRITATI /2/ 35888 M 500132
91	33	PBI09C004-BC-DH70	BERKUT/2/BERKUT / 35883 M500110
92	158	PBI09C026-BC-DH101	WAXWING*2/KIRITATI /3/WAXWING*2/KIRITATI /2/ 35888 M 500132
93	179	PBI09C028-BC-DH46	WAXWING*2/KIRITATI /3/WAXWING*2/KIRITATI /2/ 35880 M C18644
94	141	PBI09C026-BC-DH51	WAXWING*2/KIRITATI /3/WAXWING*2/KIRITATI /2/ 35888 M 500132
95	220	PBI09C035-BC-DH23	PBW502 /2/PBW502 / 19385 KC75
96	252	PBI09C039-BC-DH52	PBW550 /2/PBW550 / 18343 KC75
97	54	PBI09C008-BC-DH3	SOKOLL/2/SOKOLL / 35883 M500110
98	301	PBI09C048-BC-DH1	DBW17 /2/DBW17 / 21758 KC75
99	199	PBI09C034-BC-DH32	PBW502 /2/PBW502 / 21758 KC75
100	228	PBI09C035-BC-DH40	PBW502 /2/PBW502 / 19385 KC75
101	249	PBI09C039-BC-DH42	PBW550 /2/PBW550 / 18343 KC75
102	221	PBI09C035-BC-DH29	PBW502 /2/PBW502 / 19385 KC75
103	197	PBI09C034-BC-DH26	PBW502 /2/PBW502 / 21758 KC75
104	266	PBI09C039-BC-DH89	PBW550 /2/PBW550 / 18343 KC75
105	138	PBI09C026-BC-DH48	WAXWING*2/KIRITATI /3/WAXWING*2/KIRITATI /2/ 35888 M 500132
106	245	PBI09C039-BC-DH13	PBW550 /2/PBW550 / 18343 KC75
107	132	PBI09C026-BC-DH35	WAXWING*2/KIRITATI /3/WAXWING*2/KIRITATI /2/ 35888 M 500132
108	222	PBI09C035-BC-DH30	PBW502 /2/PBW502 / 19385 KC75
109	119	PBI09C026-BC-DH12	WAXWING*2/KIRITATI /3/WAXWING*2/KIRITATI /2/ 35888 M 500132
110	212	PBI09C035-BC-DH10	PBW502 /2/PBW502 / 19385 KC75
111	130	PBI09C026-BC-DH32	WAXWING*2/KIRITATI /3/WAXWING*2/KIRITATI /2/ 35888 M 500132
112	203	PBI09C034-BC-DH40	PBW502 /2/PBW502 / 21758 KC75
113	131	PBI09C026-BC-DH33	WAXWING*2/KIRITATI /3/WAXWING*2/KIRITATI /2/ 35888 M 500132
114	205	PBI09C034-BC-DH42	PBW502 /2/PBW502 / 21758 KC75
115	255	PBI09C039-BC-DH57	PBW550 /2/PBW550 / 18343 KC75
116	127	PBI09C026-BC-DH25	WAXWING*2/KIRITATI /3/WAXWING*2/KIRITATI /2/ 35888 M 500132
117	184	PBI09C028-BC-DH56	WAXWING*2/KIRITATI /3/WAXWING*2/KIRITATI /2/ 35880 M C18644
118	175	PBI09C028-BC-DH40	WAXWING*2/KIRITATI /3/WAXWING*2/KIRITATI /2/ 35880 M C18644
119	97	PBI09C009-BC-DH96	SOKOLL/2/SOKOLL / 35888 M 500132
120	214	PBI09C035-BC-DH14	PBW502 /2/PBW502 / 19385 KC75
121	153	PBI09C026-BC-DH89	WAXWING*2/KIRITATI /3/WAXWING*2/KIRITATI /2/ 35888 M 500132
122	218	PBI09C035-BC-DH18	PBW502 /2/PBW502 / 19385 KC75
123	142	PBI09C026-BC-DH53	WAXWING*2/KIRITATI /3/WAXWING*2/KIRITATI /2/ 35888 M 500132
124	22	PBI09C004-BC-DH33	BERKUT/2/BERKUT / 35883 M500110
125	178	PBI09C028-BC-DH45	WAXWING*2/KIRITATI /3/WAXWING*2/KIRITATI /2/ 35880 M C18644
126	121	PBI09C026-BC-DH14	WAXWING*2/KIRITATI /3/WAXWING*2/KIRITATI /2/ 35888 M 500132
127	125	PBI09C026-BC-DH20	WAXWING*2/KIRITATI /3/WAXWING*2/KIRITATI /2/ 35888 M 500132
128	29	PBI09C004-BC-DH60	BERKUT/2/BERKUT / 35883 M500110
129	128	PBI09C026-BC-DH26	WAXWING*2/KIRITATI /3/WAXWING*2/KIRITATI /2/ 35888 M 500132
130	293	PBI09C045-BC-DH11	DBW16 /2/DBW16 / 18341 KC75
131	323	PBI09C048-BC-DH38	DBW17 /2/DBW17 / 21758 KC75
132	140	PBI09C026-BC-DH50	WAXWING*2/KIRITATI /3/WAXWING*2/KIRITATI /2/ 35888 M 500132
133	173	PBI09C028-BC-DH35	WAXWING*2/KIRITATI /3/WAXWING*2/KIRITATI /2/ 35880 M C18644
134	274	PBI09C043-BC-DH39	DBW16 /2/DBW16 / 21758 KC75
135	126	PBI09C026-BC-DH24	WAXWING*2/KIRITATI /3/WAXWING*2/KIRITATI /2/ 35888 M 500132
136	95	PBI09C009-BC-DH93	SOKOLL/2/SOKOLL / 35888 M 500132
137	112	PBI09C026-BC-DH4	WAXWING*2/KIRITATI /3/WAXWING*2/KIRITATI /2/ 35888 M 500132
138	81	PBI09C009-BC-DH65	SOKOLL/2/SOKOLL / 35888 M 500132

139	239	PBI09C038-BC-DH14	PBW550 /2/PBW550 / 18293 KC75
140	202	PBI09C034-BC-DH38	PBW502 /2/PBW502 / 21758 KC75
141	123	PBI09C026-BC-DH16	WAXWING*2/KIRITATI /3/WAXWING*2/KIRITATI /2/ 35888 M 500132
142	297	PBI09C045-BC-DH19	DBW16 /2/DBW16 / 18341 KC75
143	248	PBI09C039-BC-DH37	PBW550 /2/PBW550 / 18343 KC75
144	59	PBI09C009-BC-DH9	SOKOLL/2/SOKOLL / 35888 M 500132
145	236	PBI09C038-BC-DH11	PBW550 /2/PBW550 / 18293 KC75
146	120	PBI09C026-BC-DH13	WAXWING*2/KIRITATI /3/WAXWING*2/KIRITATI /2/ 35888 M 500132
147	162	PBI09C028-BC-DH15	WAXWING*2/KIRITATI /3/WAXWING*2/KIRITATI /2/ 35880 M C18644
148	269	PBI09C043-BC-DH21	DBW16 /2/DBW16 / 21758 KC75
149	129	PBI09C026-BC-DH30	WAXWING*2/KIRITATI /3/WAXWING*2/KIRITATI /2/ 35888 M 500132
150	147	PBI09C026-BC-DH68	WAXWING*2/KIRITATI /3/WAXWING*2/KIRITATI /2/ 35888 M 500132
151	24	PBI09C004-BC-DH36	BERKUT/2/BERKUT / 35883 M500110
152	240	PBI09C038-BC-DH15	PBW550 /2/PBW550 / 18293 KC75
153	324	PBI09C051-BC-DH3	DBW17 /2/DBW17 / 18341 KC75
154	180	PBI09C028-BC-DH47	WAXWING*2/KIRITATI /3/WAXWING*2/KIRITATI /2/ 35880 M C18644
155	76	PBI09C009-BC-DH49	SOKOLL/2/SOKOLL / 35888 M 500132
156	321	PBI09C048-BC-DH36	DBW17 /2/DBW17 / 21758 KC75
157	172	PBI09C028-BC-DH34	WAXWING*2/KIRITATI /3/WAXWING*2/KIRITATI /2/ 35880 M C18644
158	265	PBI09C039-BC-DH87	PBW550 /2/PBW550 / 18343 KC75
159	92	PBI09C009-BC-DH82	SOKOLL/2/SOKOLL / 35888 M 500132
160	111	PBI09C026-BC-DH3	WAXWING*2/KIRITATI /3/WAXWING*2/KIRITATI /2/ 35888 M 500132
161	209	PBI09C035-BC-DH6	PBW502 /2/PBW502 / 19385 KC75
162	286	PBI09C043-BC-DH63	DBW16 /2/DBW16 / 21758 KC75
163	151	PBI09C026-BC-DH86	WAXWING*2/KIRITATI /3/WAXWING*2/KIRITATI /2/ 35888 M 500132
164	254	PBI09C039-BC-DH55	PBW550 /2/PBW550 / 18343 KC75
165	166	PBI09C028-BC-DH22	WAXWING*2/KIRITATI /3/WAXWING*2/KIRITATI /2/ 35880 M C18644
166	305	PBI09C048-BC-DH7	DBW17 /2/DBW17 / 21758 KC75
167	282	PBI09C043-BC-DH59	DBW16 /2/DBW16 / 21758 KC75
168	243	PBI09C039-BC-DH2	PBW550 /2/PBW550 / 18343 KC75
169	150	PBI09C026-BC-DH85	WAXWING*2/KIRITATI /3/WAXWING*2/KIRITATI /2/ 35888 M 500132
170	234	PBI09C038-BC-DH5	PBW550 /2/PBW550 / 18293 KC75
171	176	PBI09C028-BC-DH41	WAXWING*2/KIRITATI /3/WAXWING*2/KIRITATI /2/ 35880 M C18644
172	115	PBI09C026-BC-DH7	WAXWING*2/KIRITATI /3/WAXWING*2/KIRITATI /2/ 35888 M 500132
173	23	PBI09C004-BC-DH34	BERKUT/2/BERKUT / 35883 M500110
174	77	PBI09C009-BC-DH51	SOKOLL/2/SOKOLL / 35888 M 500132
175	244	PBI09C039-BC-DH12	PBW550 /2/PBW550 / 18343 KC75
176	250	PBI09C039-BC-DH43	PBW550 /2/PBW550 / 18343 KC75
177	204	PBI09C034-BC-DH41	PBW502 /2/PBW502 / 21758 KC75
178	117	PBI09C026-BC-DH10	WAXWING*2/KIRITATI /3/WAXWING*2/KIRITATI /2/ 35888 M 500132
179	264	PBI09C039-BC-DH86	PBW550 /2/PBW550 / 18343 KC75
180	259	PBI09C039-BC-DH70	PBW550 /2/PBW550 / 18343 KC75
181	152	PBI09C026-BC-DH87	WAXWING*2/KIRITATI /3/WAXWING*2/KIRITATI /2/ 35888 M 500132
182	118	PBI09C026-BC-DH11	WAXWING*2/KIRITATI /3/WAXWING*2/KIRITATI /2/ 35888 M 500132
183	311	PBI09C048-BC-DH15	DBW17 /2/DBW17 / 21758 KC75
184	181	PBI09C028-BC-DH50	WAXWING*2/KIRITATI /3/WAXWING*2/KIRITATI /2/ 35880 M C18644
185	75	PBI09C009-BC-DH48	SOKOLL/2/SOKOLL / 35888 M 500132
186	124	PBI09C026-BC-DH19	WAXWING*2/KIRITATI /3/WAXWING*2/KIRITATI /2/ 35888 M 500132
187	207	PBI09C035-BC-DH1	PBW502 /2/PBW502 / 19385 KC75
188	157	PBI09C026-BC-DH97	WAXWING*2/KIRITATI /3/WAXWING*2/KIRITATI /2/ 35888 M 500132
189	170	PBI09C028-BC-DH31	WAXWING*2/KIRITATI /3/WAXWING*2/KIRITATI /2/ 35880 M C18644
190	267	PBI09C043-BC-DH4	DBW16 /2/DBW16 / 21758 KC75
191	322	PBI09C048-BC-DH37	DBW17 /2/DBW17 / 21758 KC75
192	219	PBI09C035-BC-DH19	PBW502 /2/PBW502 / 19385 KC75
193	211	PBI09C035-BC-DH9	PBW502 /2/PBW502 / 19385 KC75
194	144	PBI09C026-BC-DH57	WAXWING*2/KIRITATI /3/WAXWING*2/KIRITATI /2/ 35888 M 500132
195	116	PBI09C026-BC-DH9	WAXWING*2/KIRITATI /3/WAXWING*2/KIRITATI /2/ 35888 M 500132
196	543	Sunlin	-

Supplementary Table 2; Parameters of the logistic growth curves fitted to main spike dry weight (MSDW) data in Supplementary Figure 5 for two wheat genotypes (G1, putative heat tolerant; G2, putative heat susceptible) in response to day/night temperatures of CET1 (control, (22/14 °C)), CET2 (30/20 °C from heading), and CET3 (30/20 °C from anthesis). Where Y_{max} is maximum MSDW, Y_0 is starting MSDW at anthesis, k is the rate constant and Xint is the first inflection point. GxT, genotype by treatment interaction.

GxT	Y_{max} (g)	Y_0 (g)	k (°Cd^{-1} x 10^{-3})	Xint (°Cd^{-1})	R^2
G1CET1	7.84	0.89	3.9	253.3	0.94
G2CET1	5.45	0.89	4.2	235.3	0.91
G1CET2	3.45	0.89	4.6	218.5	0.94
G2CET2	2.66	0.89	4.9	204.3	0.92
G1CET3	4.06	0.89	4.4	228.9	0.96
G2CET3	3.13	0.89	4.1	242.8	0.94

Supplementary Table 3; Parameters of the asymmetric Gompertz curves fitted to SPAD values in the glasshouse experiment of Figure 3 (GH) for two wheat genotypes (G1, putative heat tolerant; G2, putative heat susceptible) in response to day/night temperatures of CET1 (control, (22/14 °C)), CET2 (30/20 °C from heading), and CET3 (30/20 °C from anthesis). Where b, c, d and e are the slope around the inflection point, lower and upper asymptotes, and the midway point between c and d. The lower asymptote was constrained to zero and hence, is not included.

GxT	b (SPAD °Cd^{-1})	d (°Cd^{-1})	e (°Cd^{-1})	R^2
G1CET1	-0.009682	52.72	367.1	0.9756
G2CET1	-0.01165	51.82	354	0.9787
G1CET2	-0.00553	53.02	297.6	0.9785
G2CET2	-0.006232	54.85	222.3	0.9841
G1CET3	-0.005336	53.39	311.2	0.9843
G2CET3	-0.006148	53.78	256.3	0.9825

Supplementary Table 4; Parameters of the best fit linear regression curves fitted to green leaf area (GLA) overtime in the glasshouse experiment of Supplementary Figure 6 for two wheat genotypes (G1, putative heat tolerant; G2, putative heat susceptible) in response to day/night temperatures of CET1 (control, (22/14 °C)), CET2 (30/20 °C from heading), and CET3 (30/20 °C from anthesis).

GxT	GLA Intercept (cm^2)	Slope (cm^2 °Cd^{-1})
G1T1	1028.1	-1.133
G2T1	1002.2	-1.300
G1T2	914.1	-1.211
G2T2	844.9	-1.197
G1T3	921.5	-1.130
G2T3	913.4	-1.274

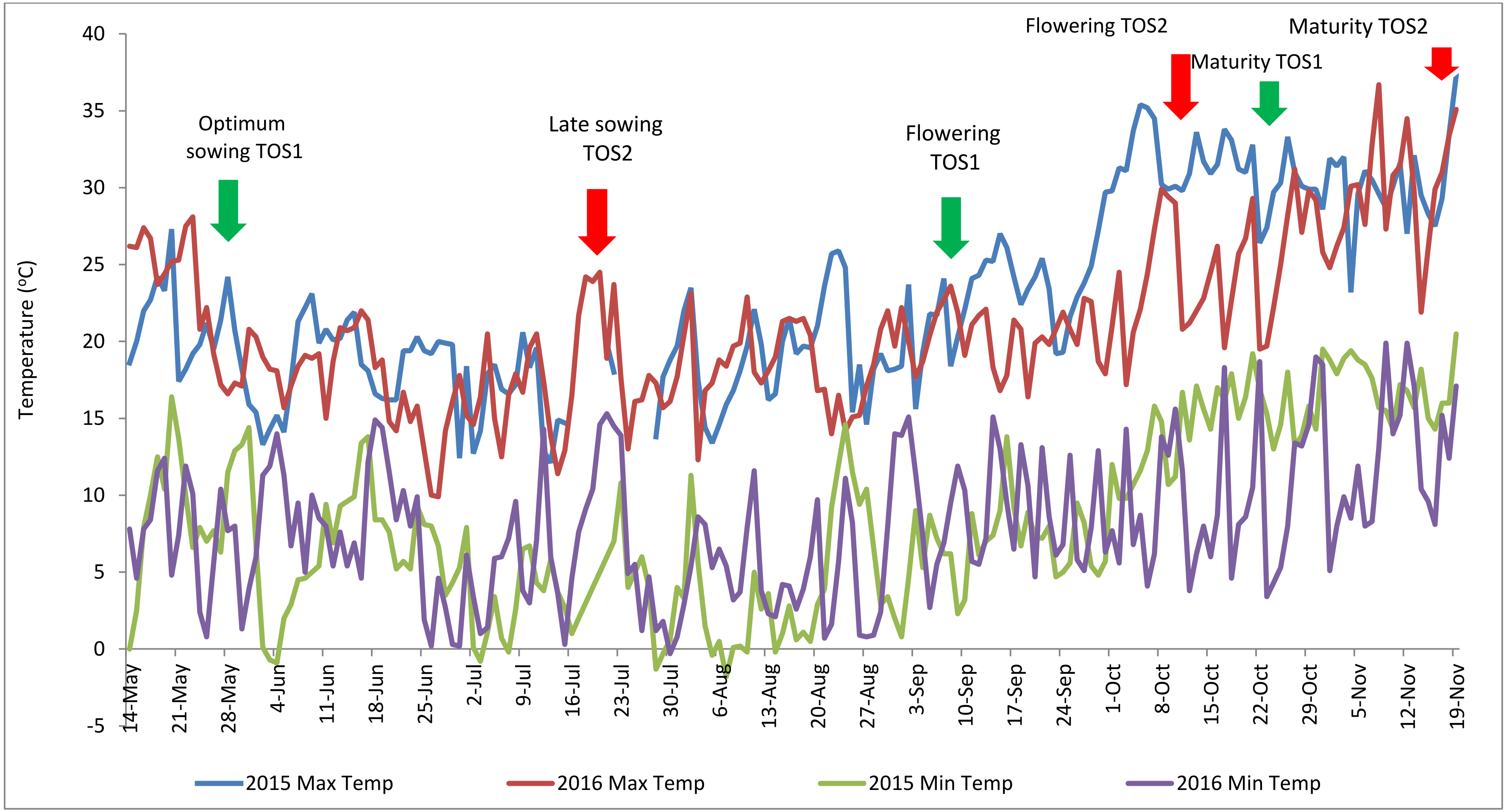

Supplementary Figure 1. Daily maximum and minimum temperature during crop growth (including both times of sowing (TOS)) in the 2015 and 2016 field trials at Narrabri. The arrows indicate timing of sowing, and average reproductive stage and physiological maturity for the optimal (TOS1) and late (TOS2) sowing dates.

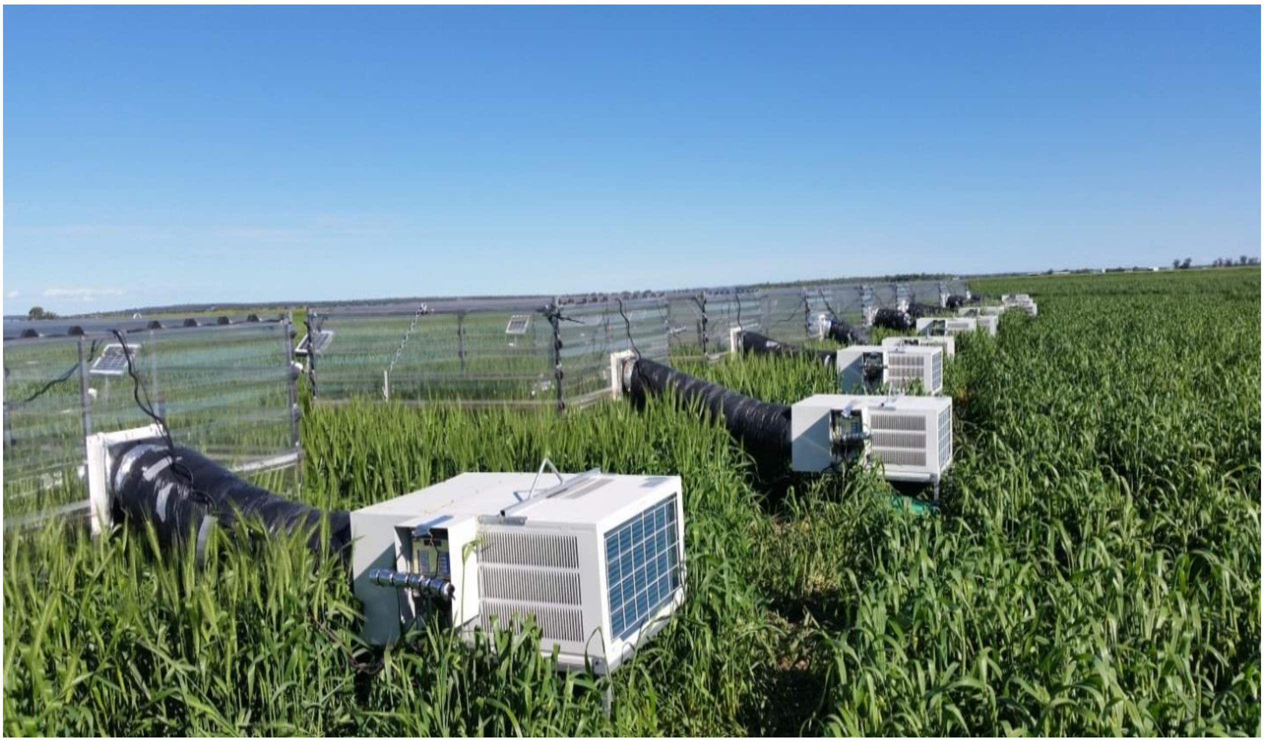

Supplementary Figure 2. Field view of the chambers used to impose heat stress at anthesis during 2016. Set temperatures were achieved using reverse cycle air conditioners (3.9 kW heating and 3.6 kW cooling) powered by heavy voltage generators (15 kVA 3 phase) and controlled using SPLAT (HVAC26A) controllers attached to each unit. Weather-proof temperature and humidity data loggers (HOBO® U23, Pro v2) were used to monitor conditions in each chamber.

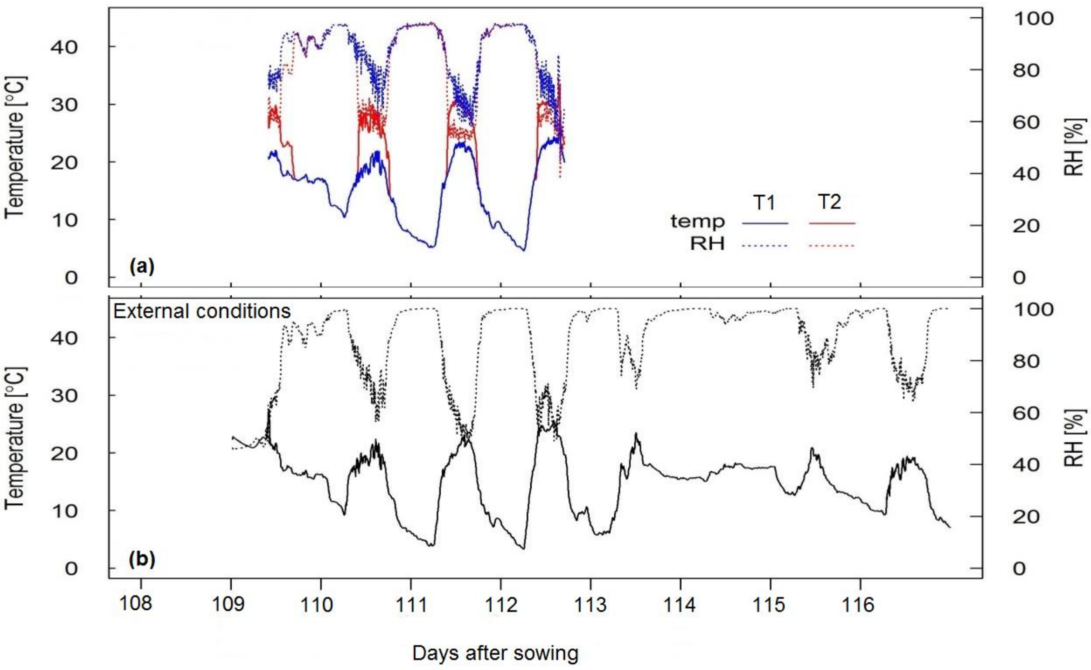

Supplementary Figure 3. Air temperature and relative humidity (RH) inside the heat chambers for the ambient (T1) and heated (T2) treatments (a) compared with external ambient conditions (b). Solid lines represent temperature and dashed lines represent RH.

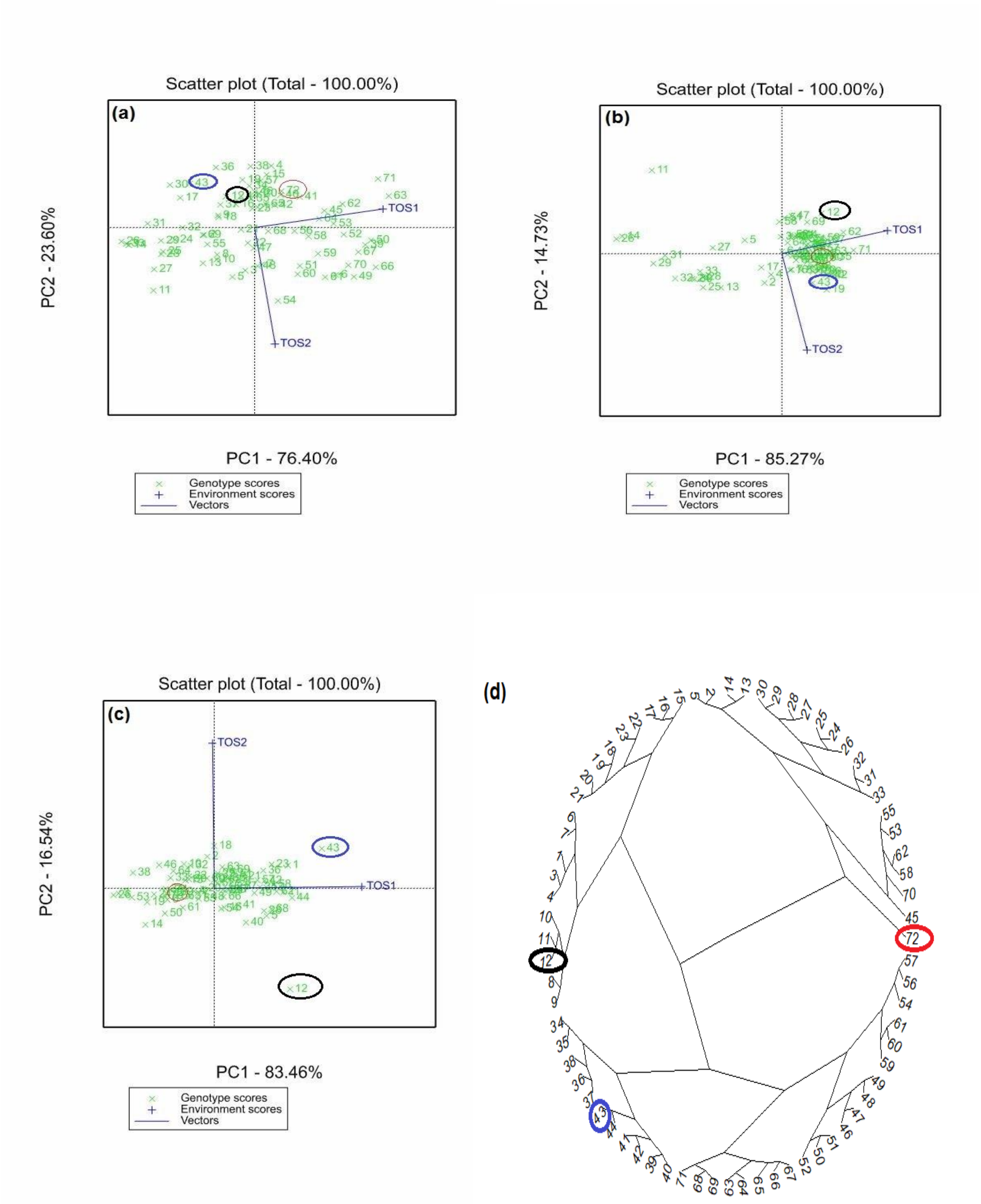

Supplementary Figure 4; Comparison of recurrent parent PBW 502 ((#72 encircled red) and progenies including stable G1 (#43 encircled blue) and unstable G2 (#12 encircled black) emmer derived field selected lines based on mean performance and stability across the two environments i.e. normal and heat stressed for (a) screening percentages, (b) TKW, (c) and grain yield, and (d) dendrogram demonstrating the genetic distance between the recurrent hexaploid parents and progenies (n = 72). PBW502, G1 and G2 emmer derived lines are indicated with the same coloured circles as in the GGE biplots.

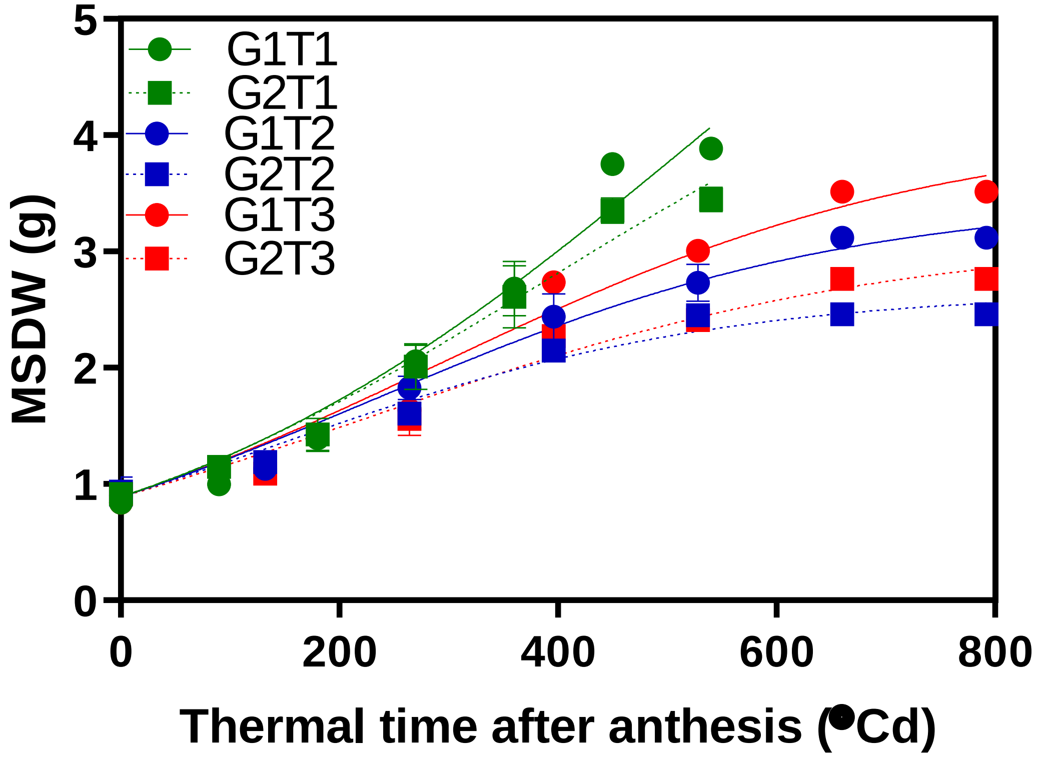

Supplementary Figure 5. Change in mean main spike dry weight (MSDW) over time for two wheat genotypes (G1, putative heat tolerant (circles and solid lines); G2, putative heat susceptible (squares and dashed lines)) in response to day/night temperatures of T1 (control, (22/14 °C)), T2 (30/20 °C from heading), and T3 (30/20 °C from anthesis) under controlled environment conditions. Error bars represent SEM, n = 4. The parameters for the logistic growth curves best fit to the data are shown in Supplementary Table 2.

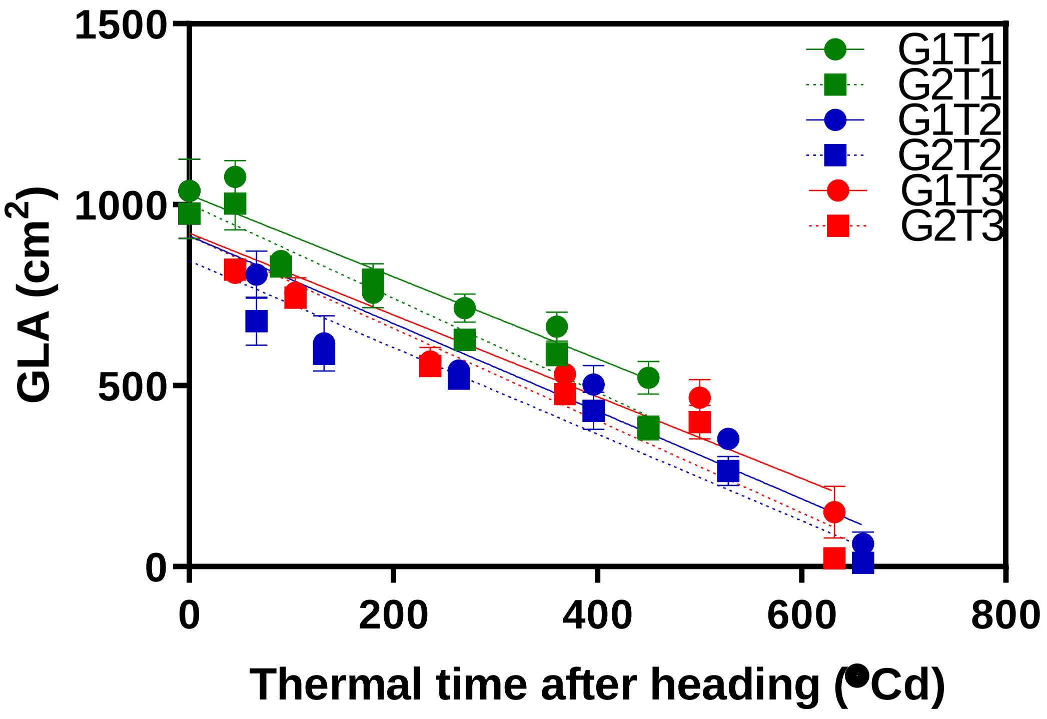

Supplementary Figure 6. Change in mean green leaf area (GLA) over time for two wheat genotypes (G1, putative heat tolerant (circles and solid lines); G2, putative heat susceptible (squares and dashed lines)) in response to day/night temperatures of T1 (control, (22/14 °C)), T2 (30/20 °C from heading), and T3 (30/20 °C from anthesis) under controlled environment conditions (GH). Error bars represent ±1 SEM, n = 4. Data were best fit by linear regression with the parameters shown in Supplementary Table 4.

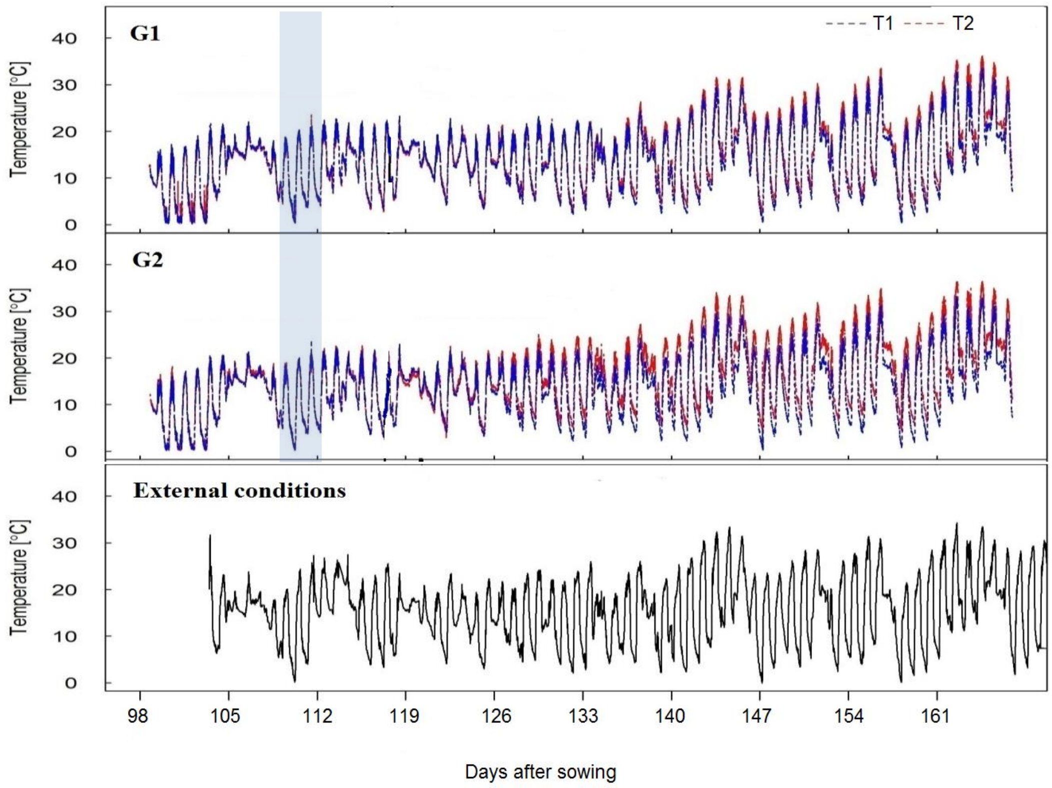

Supplementary Figure 7. Canopy temperature (°C) of genotypes assessed from booting to physiological maturity exposed to ambient (T1, blue lines) and heated (T2, red lines) chambers for 4 days from anthesis (110 days after sowing). The shaded portion in the upper two panels indicates when the heat chambers were applied.
